# Coexposure to extreme heat, wildfire burn zones, and wildfire smoke in the Western US from 2006 to 2020

**DOI:** 10.1126/sciadv.adq6453

**Published:** 2025-04-30

**Authors:** Jie K. Hu, Ana Trišović, Ankita Bakshi, Danielle Braun, Francesca Dominici, Joan A. Casey

**Affiliations:** ^1^ Department of Statistics, The Ohio State University College of Arts and Sciences, Cockins Hall, 1958 Neil Ave, Columbus, OH, USA.; ^2^ Computer Science and Artificial Intelligence Laboratory (CSAIL), Massachusetts Institute of Technology, 32 Vassar St., Cambridge, MA, USA.; ^3^ Department of Biostatistics, Harvard T.H. Chan School of Public Health, 677 Huntington Avenue, Boston, MA, USA.; ^4^ Esri, 380 New York Street, Redlands, CA, USA.; ^5^ Department of Data Science, Dana Farber Cancer Institute, 3 Blackfan Circle, Boston, MA, USA.; ^6^ Department of Environmental and Occupational Health Sciences, University of Washington School of Public Health, 3980 15th Avenue Northeast, Seattle, WA, USA.

## Abstract

Climate change drives three heat-related hazards: extreme heat (EH), wildfire burn zones (WFBZs), and wildfire smoke (WFS). Using daily census tract–level data from 2006 to 2020, we investigated when, where, and whom these hazards coexposed in 11 Western US states. Among 18,106 tracts, at least one hazard occurred an average of 32 days (581,867 tract-days) annually. EH-WFS coexposure increased over the study period and was the most frequent coexposure (annual average of 38,218 tract-days). EH-WFS–affected regions varied year to year. WFBZ-involved coexposures were spatially confined and did not increase over time. On average, the most tract-days of EH-WFBZ-WFS coexposure took place in California, Arizona, and Oregon. Among census tracts most exposed to EH-WFBZ-WFS, populations disproportionately consisted of people of older age, with disabilities, and living in poverty. American Indian and Alaska Native individuals disproportionately faced all coexposures. As climate change accelerates, tracking coexposure to multiple hazards can help target resources to protect health.

## Teaser

Extreme heat, wildfire smoke, and their coexposure increased during 2006–2020 in the Western US.

## INTRODUCTION

As climate change advances, human populations will have to contend not just with single hazards, such as wildfires, but increasingly with co-occurring events, such as wildfires and extreme heat (EH). This has already begun globally. For example, during Australia’s bushfire crisis (2019) (*1*) and in Siberia (2019 and 2020), California (2017, 2018, and 2020) (*2*–*4*), Greece (2023) (*5*), and Italy (1991–2009) (*6*), wildfires happened concurrently with EH. These climate hazards are interconnected. EH can increase the likelihood of wildfire by drying vegetation that can fuel the fires (*1*, *4*, *6*, *7*). Severe wildfires can also increase local temperatures.

Wildfires present two distinct hazards: wildfire burn zones (WFBZs) and wildfire smoke (WFS) exposure. Those living proximate to WFBZs, in addition to WFS exposure, can also experience financial loss, trauma, and physical health effects (*8*–*12*). WFS exposure, broadly affecting people living farther from the burn zone, is associated with an increased risk of respiratory and cardiovascular health care visits and adverse birth outcomes (*13*–*16*). We consider WFBZ and WFS exposure separately. WFBZs affect much smaller areas more intensely, and WFS can spread across hundreds of kilometers. We assess coexposure patterns to WFBZ-EH compared to WFS-EH.

EH and wildfires cause substantial economic damage (*17*). The US may lose approximately $100 billion annually from heat-induced lost labor productivity by 2050 (*18*), and the economic burden of wildfires, inclusive of health impacts, already tops at least $70 billion annually (*19*). The 2018 California wildfires alone caused $149 billion ($126 billion to $193 billion) in total damages, including $32 billion ($13 billion to $76 billion) in health costs estimated via mortality, medical expenses, and work time lost (*20*). However, different sociodemographic groups often do not share the economic burdens equally. This may be related to where individuals of various socioeconomic groups reside in relation to hazards and variation in preparedness and ability to recover from exposure to hazards. For example, Black and Hispanic workers disproportionately bear heat-induced lost labor productivity (*18*), and communities of color often experience disproportionate climate change–related health effects (*21*). Likewise, EH and wildfires may more powerfully affect the health of specific populations, for example, those with preexisting health conditions, older adults, or children (*22*, *23*).

Now, no studies evaluate the spatiotemporal distribution of coexposure to EH, WFBZ, and WFS. The limited literature on dual coexposure (*24*–*26*) has focused on coexposure for a specific event, period, or region. For instance, Rosenthal *et al.* (*25*) investigated population coexposure to EH and WFS in California during the wildfire season in 2020, finding 16.5 million people experienced a coexposure event. Austin *et al.* (*24*) found that Washington State counties with more agricultural workers had the most dual heat and wildfire season air pollution exposures between 2010 and 2018. In addition, most coexposure studies have focused on the county (*24*) or the zip code level (*27*), except for Rosenthal *et al.* (*25*), who used a 3-km spatial resolution in 2020 in their EH-WFS study in California. Using fine-resolution data nationwide over a long-term period may uncover different rates of co-occurrence, different estimates of the population exposed, or issues of environmental injustice.

Here, we conceptualized and formulated metrics to evaluate the coexposure risk to EH, WFBZ, and WFS at the census tract level in 11 Western US states from 2006 to 2020. We assembled high-resolution satellite and model data on these three hazards to characterize the spatiotemporal distribution and change in the coexposure to these three hazards over the study period. We also evaluated potential disproportionate exposure by racial/ethnic and socioeconomic groups and identified priority census tracts for action. Our data and code are available on Harvard Dataverse.

## RESULTS

### Study scope

The study spanned 2006–2020 in 18,106 census tracts across 414 counties in 11 contiguous Western US states—Arizona, California, Colorado, Idaho, Montana, Nevada, New Mexico, Oregon, Utah, Washington, and Wyoming—an area containing 75,506,421 people. We identified the presence of and coexposure to three climate hazards at the census tract level: EH, WFBZ, and WFS. We defined an EH tract-day based on the gridMET maximum air temperature dataset when the maximum daily temperature both equaled and exceeded the relative threshold (warm season 95th percentile) and the absolute threshold [90°F (32.22°C)] in a census tract. We used Moderate Resolution Imaging Spectroradiometer (MODIS) C6.1 MCD41A1 active fire/hot spot data to define a WFBZ tract-day as any wildfire burning on a day in a census tract. We obtained daily census tract WFS PM_2.5_ (fine particulate matter with a diameter of 2.5 micrometers or less) exposure estimates from a previously developed machine learning model (*28*). A WFS tract-day was defined as tract-level daily WFS PM_2.5_ exceeding 0 μg/m^3^. When a hazard occurred in a tract, we assumed that it exposed everyone there. We defined coexposures based on two or more hazards occurring on the same tract-day.

### Summary of total climate hazard occurrences

From 2006 to 2020, the 18,106 census tracts in the Western US experienced one or more of the three climate hazards for an annual average of 581,867 tract-days and an annual average of 32 days per tract. The 11-state study area experienced an annual average of 133,714 EH tract-days, 7709 WFBZ tract-days, and 481,564 WFS tract-days (Table 1). Assuming that every person in an exposed tract experienced the hazard, these tract-level exposures translated into an annual average of 31,171 person-days of EH exposure, 1394 person-days of WFBZ exposure, and 109,147 person-days of WFS exposure per census tract. Over the 15-year study period, the Western US had an annual average of 2.4 billion person-days where at least one of the three climate hazards occurred and 511,904 person-days where all three occurred (table S1).

**Table 1.** Annual average prevalence of census tract–level climate hazards in 11 Western US states, 2006–2020.

	Average exposure tract-days per year	Average exposure days per census tract per year
	Mean (SD*)	
Single hazards		
EH^†^	133,714 (47,400)	7.4 (3.4)
WFBZ^‡^	7,709 (1,219)	0.4 (2.5)
WFS^§^	481,564 (259,819)	26.6 (10.7)
EH, WFBZ, or WFS	581,806 (268,376)	32.1 (10.4)
Co-occurring hazards		
EH and WFBZ	209 (107)	0.0 (0.1)
EH and WFS	38,214 (30,679)	2.1 (1.1)
WFBZ and WFS	2,913 (984)	0.2 (1.1)
EH, WFBZ, and WFS	154 (99)	0.0 (0.1)

*We calculate the SD of tract-days across the census tracts, and for the average exposure days per census tract, we compute the SD across years.

†Defined on the basis of the local maximum daily temperature equaling or exceeding the warm season 95th percentile and 90°F (32.22°C).

‡Defined as an active fire/hot spot identified by MODIS C6.1 MCD41A1 in the census tract.

§Defined as daily WFS PM_2.5_ exceeding 0 μg/m^3^.

Coexposure to multiple hazards occurred frequently. EH-WFS was the most common, with an annual average exposure of 38,214 tract-days and 8788 person-days per tract (Table 1 and table S1). In comparison, we observed 2913 tract-days of WFBZ-WFS exposure and 209 tract-days of EH-WFBZ exposure annually. In a sensitivity analysis, by changing the threshold for defining a WFS tract-day to WFS PM_2.5_ > 5 μg/m^3^ from >0 μg/m^3^, the number of annual average WFS tract-days was reduced by two-thirds from 481,564 to 161,283 (Table 1 and table S2). Likewise, annual average EH-WFS coexposure was reduced from 38,214 to 14,662 tract-days, and WFBZ-WFS coexposure was reduced from 2913 to 1285 tract-days (Table 1 and table S2). In a second sensitivity analysis, we expanded the coexposure time window from the same day to 2 days. This resulted in an increase in EH-WFS (>0 μg/m^3^ threshold) coexposure from an annual average of 38,214 to 73,931 tract-days (Table 1 and table S2). WFBZ-WFS (>0 μg/m^3^ threshold) annual average exposure also increased from 2913 to 5649 tract-days. When pairing the WFS PM_2.5_ threshold (>5 μg/m^3^) with the 2-day exposure window, we identified an annual average of 28,495 EH-WFS and 2100 WFBZ-WFS coexposure tract-days per year (table S2).

### Spatial patterns

**Fig. 1.**
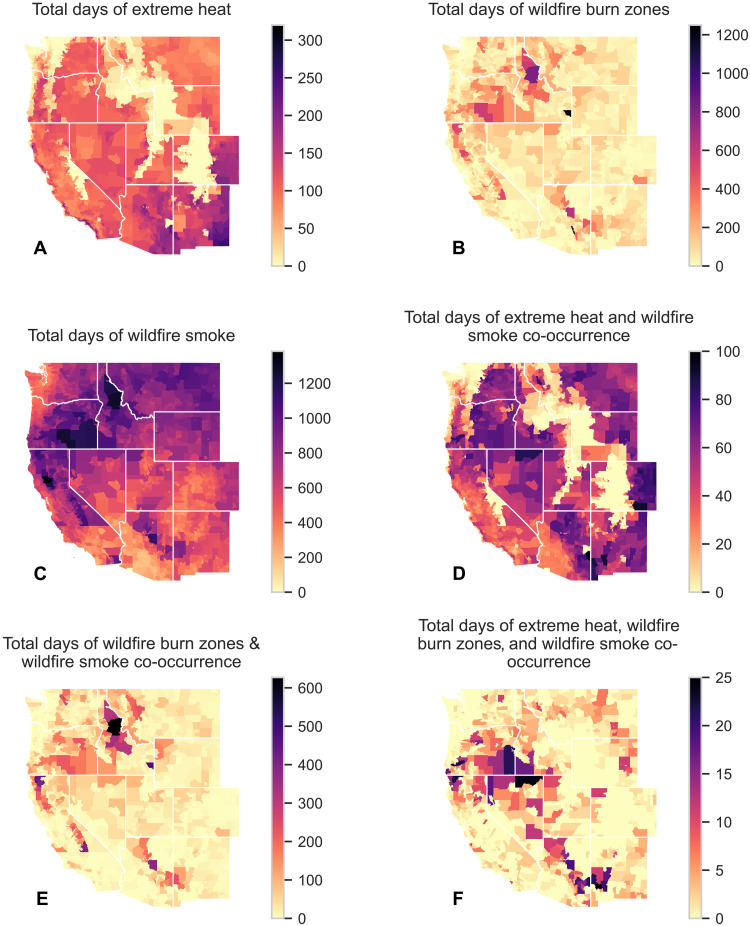
Total climate hazard days by census tract in 11 Western US states, 2006–2020. (**A**) EH; (**B**) WFBZs; (**C**) WFS; (**D**) EH and WFS; (**E**) WFBZs and WFS; and (**F**) EH, WFBZs, and WFS. A WFBZ was defined as an active fire/ hot spot identified by MODIS C6.1 MCD41A1 in the census tract. WFS was defined as daily WFS PM_2.5_ concentration over 0 μg/m^3^. Black indicates the most exposure days, and light yellow indicates the fewest. Note the difference in legend scales across the panels.

**Fig. 2.**
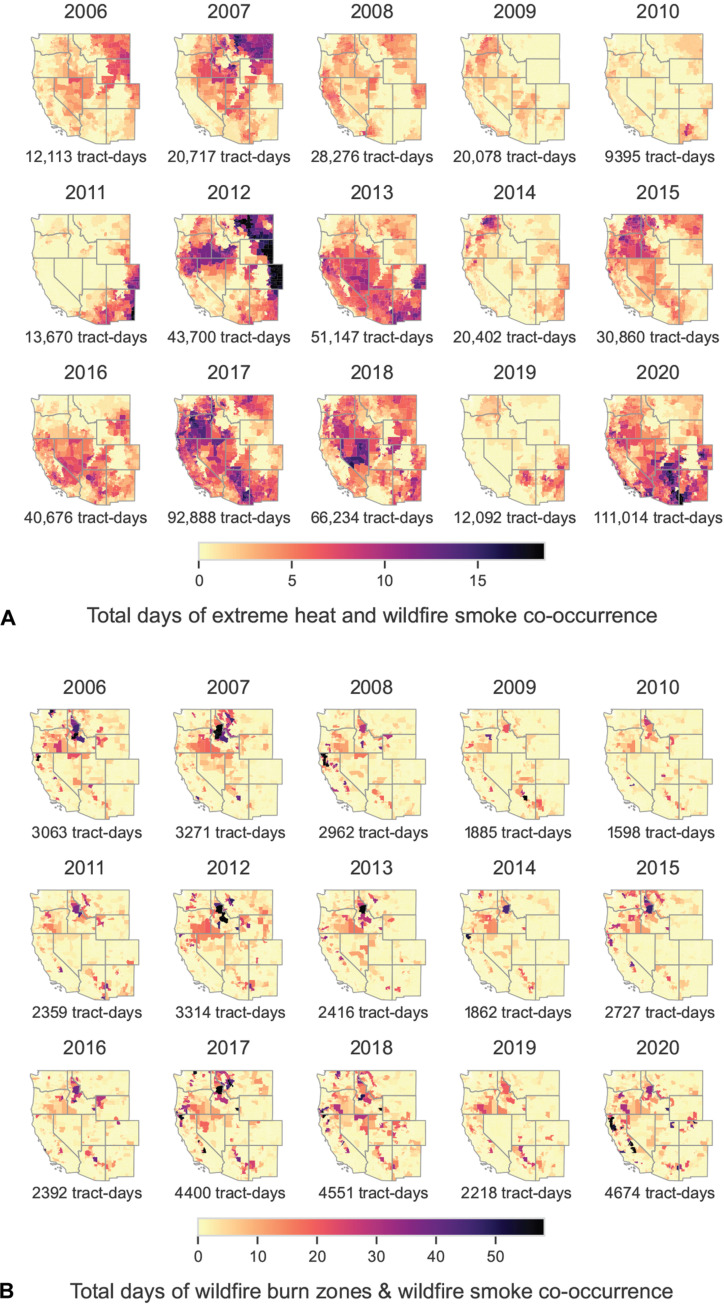
Spatial distribution of climate hazard coexposure days at the census tract–level in 11 Western US states by year, 2006–2020. (**A**) Total tract-days of EH and WFS coexposure by year; (**B**) total tract-days of WFBZ and WFS coexposure by year. Black indicates the most exposure days and light yellow the fewest.

From 2006 to 2020, single climate hazards versus co-occurring hazards exhibited different spatial patterns (Fig. 1). Specifically, New Mexico, Eastern Arizona, Coastal California, and Eastern Colorado had the most EH days (Fig. 1A), Oregon and Idaho had the most WFBZ days (Fig. 1B), and California, Oregon, Eastern Washington, Idaho, Montana, and Wyoming had the most WFS days (Fig. 1C). EH-WFS coexposure affected every Western state (Fig. 1D). WFBZ coexposure was more confined (Figs. 1E and 2B and fig. S3, B and C), with a persistent WFBZ-WFS hot spot in Idaho (Fig. 2B). California’s Sierra Nevada mountains also frequently experienced WFBZ-WFS coexposure (Fig. 2B). EH-WFBZ-WFS coexposure largely occurred in Southern Oregon, Northern Nevada, and parts of Arizona and New Mexico (Fig. 1F). EH-WFBZ coexposure followed a similar spatial pattern to that of EH-WFBZ-WFS (Fig. 1F and fig. S1).

In examining Fig. 1A, the California coastline appeared to have more EH exposure than noncoastline areas. However, we found no quantitative difference in the average number of EH days between coastal and noncoastal census tracts (fig. S2).

WFBZ (Fig. 1B) and WFS (Fig. 1C) exposures showed distinct spatial distributions. For instance, Montana recorded an average of 751 WFS days but only 29 WFBZ days per census tract from 2006 to 2020. Similarly, Colorado registered an average of 456 WFS days while only experiencing 3.5 WFBZ days per census tract. In a sensitivity analysis, we observed dampened but similar spatial patterns when using a WFS threshold of >5 μg/m^3^. This phenomenon is likely due to the midlatitude jet stream moving from west to east, which has the greatest impact on Montana and Colorado.

We also calculated state-level single and co-occurring exposures by aggregating census tract–level exposures to the state level. Compared to tract-level exposure days reported in Figs. 1 and 2, this exposure metric considers the number of affected census tracts in each state. The most EH exposure took place in California (67,866 tract-days per year and 298 million person-days per year), Arizona (15,832 tract-days per year and 64 million person-days per year), and Colorado (11,950 tract-days per year and 48 million person-days per year) (Table 2 and table S3). California also led in total WFBZ exposure (2334 tract-days per year and 8.7 million person-days per year), followed by Oregon (1068 tract-days per year and 3.5 million person-days per year) and Idaho (892 tract-days per year and 2.9 million person-days per year). California, Washington, and Colorado had the most annual average WFS exposure with 232,190, 52,120, and 43,976 tract-days per year, respectively, or 994, 218, and 173 million person-days of exposure per year, respectively.

**Table 2.** Annual average statewide tract-days of exposure to climate hazards in 11 Western US states, 2006–2020.

	Annual average statewide tract-days of exposure						
State	EH*	WFBZ^†^	WFS^‡^	EH-WFBZ	EH-WFS	WFBZ-WFS	EH-WFBZ-WFS
AZ	15,832	819	25,633	39	3532	247	25
CA	67,866	2334	232,190	48	16,677	826	35
CO	11,950	337	43,976	7	4,413	100	4
ID	2826	892	20,471	16	1,91	413	13
MT	1582	622	15,988	14	942	302	12
NM	6161	363	13,701	19	1607	116	13
NV	6427	134	16,262	11	1728	57	8
OR	8153	1068	36,194	29	3002	418	24
UT	5325	257	18,440	8	1919	98	6
WA	6755	694	52,120	13	2583	249	8
WY	836	189	6589	6	420	87	5

*Defined on the basis of the local maximum daily temperature equaling or exceeding the warm season 95th percentile and 90°F (32.22°C).

†Defined as an active fire/hot spot identified by MODIS C6.1 MCD41A1 in the census tract.

‡Defined as daily WFS PM_2.5_ exceeding 0 μg/m^3^.

California, Arizona, and Oregon had the most EH-WFBZ and the most EH-WFBZ-WFS coexposure tract-days (Table 2). Annual average person-days of EH-WFBZ-WFS coexposure totaled 139,000 in California and approximately 80,000 in Arizona and Oregon (table S3).

### Temporal patterns

For single exposures, we observed a general increase in EH (*P* = 0.02) and WFS (*P* = 0.03) tract-days from 2006 to 2020, but no similar trend emerged for WFBZ (*P* = 1.0) (fig. S3). We found an increasing trend in EH-WFS coexposure (*P* = 0.03), with more exposed tract-days from 2016 to 2020 compared to 2011–2015 and 2006–2010 (Fig. 2A). The EH-WFS–affected regions varied, with northern states more coexposed in 2006, 2007, 2012, and 2015 and southern states more coexposed in 2013, 2016, and 2020 (Fig. 2A). Similar to WFBZ, overall, WFBZ coexposures did not increase over the study period (EH-WFBZ *P* = 0.59; WFBZ-WFS *P* = 0.37) (fig. S3).

The annual prevalence of tracts experiencing any of the three hazards increased (*P* = 0.02) from 2006 to 2020 (fig. S3G), with 2017, 2018, and 2020 being the most affected. No temporal trend existed for coexposure to all three hazards (*P* = 0.46) (fig. S3H). When using a WFS PM_2.5_ threshold of >5 μg/m^3^, we, again, saw no temporal trend for EH-WFBZ-WFS (*P* = 0.37; fig. S4).

**Fig. 3.**
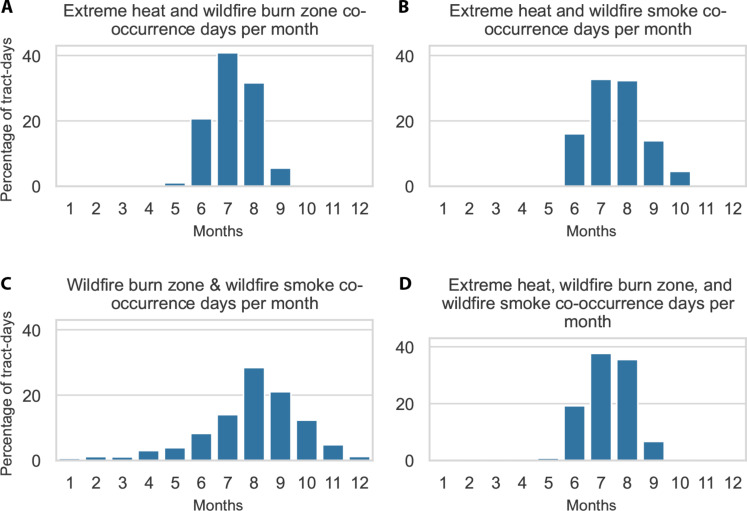
Percentage of census tract-days of coexposures by month across 11 Western US states, 2006–2020. (**A**) EH and WFBZs; (**B**) EH and WFS; (**C**) WFBZs and WFS; and (**D**) EH, WFBZs, and WFS. A WFBZ was defined as an active fire/ hot spot identified by MODIS C6.1 MCD41A1 in the census tract. WFS was defined as daily WFS PM_2.5_ exceeding 0 μg/m^3^. Percentages are calculated of total tract-days where the coexposure occurred.

Prevalence of the climate hazards fluctuated by month, with an approximately normal distribution for single EH and WFS tract-days, which peaked in the summer (fig. S5). WFBZ tract-days were more evenly distributed but peaked in late summer or early fall (fig. S5). The highest percentage of tract-days with EH-WFBZ or EH-WFS coexposure ramped up in June, peaked in July and August, and declined in September (Fig. 3). WFBZ-WFS coexposure also peaked in August but was otherwise more evenly distributed throughout the year (Fig. 3C). Coexposure to all three hazards only occurred from June to September (Fig. 3D).

**Fig. 4.**
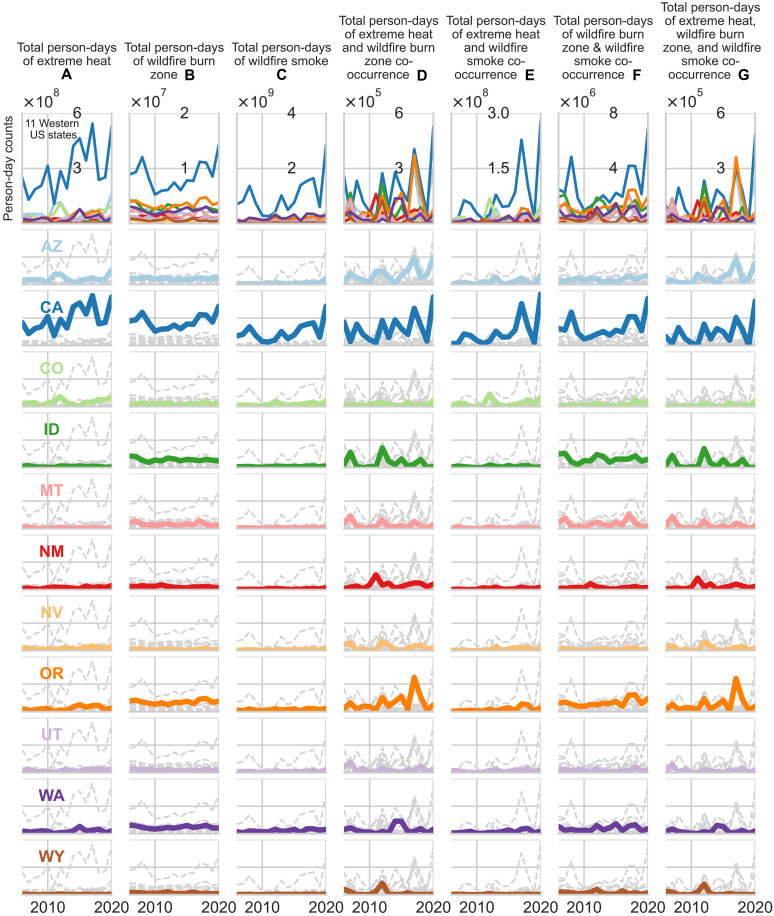
Temporal trends of climate hazard exposure and coexposure person-days in 11 Western US states, 2006–2020. The first row displays all 11 states combined, and subsequent rows represent single states, distinguished by color. Each column corresponds to an exposure or coexposure measured in person-days: (**A**) EH; (**B**) WFBZs; (**C**) WFS; (**D**) EH and WFS; (**E**) WFBZs and WFS; (**F**) EH and WFBZ; and (**G**) EH, WFBZs, and WFS. Different colored lines indicate different states: Arizona (AZ), California (CA), Colorado (CO), Idaho (ID), Montana (MT), New Mexico (NM), Nevada (NV), Oregon (OR), Utah (UT), Washington (WA), and Wyoming (WY). A WFBZ was defined as an active fire/ hot spot identified by MODIS C6.1 MCD41A1 in the census tract. WFS was defined as daily WFS PM_2.5_ exceeding 0 μg/m^3^.

Temporal patterns in climate hazard exposures differed by state (Fig. 4 and table S4). For single exposures, we observed an increasing trend in EH person-days in California (*P* = 0.02), Colorado (*P* = 0.05), New Mexico (*P* = 0.01), Nevada (*P* = 0.05), and Oregon (*P* = 0.05) from 2006 to 2020. Only New Mexico (*P* = 0.02) demonstrated an increasing trend in WFBZ person-days. We found an increasing trend in WFS person-days in Arizona (*P* = 0.004), Colorado (*P* = 0.02), Oregon (*P* = 0.04), and Washington (*P* = 0.03) and near-increasing trends in California and New Mexico (*P* = 0.06) from 2006 to 2020. For coexposures, we found a near-increasing trend of EH-WFBZ in Arizona (*P* = 0.06) and an increasing trend in EH-WFS coexposure person-days in California (*P* = 0.01), New Mexico (*P* = 0.01), and Oregon (*P* = 0.04) and a near-increasing trend in Washington (*P* = 0.06) from 2006 to 2020. Colorado alone experienced an increasing trend in WFBZ-WFS person-days (*P* = 0.01). Only Arizona had an increasing trend of EH-WFBZ-WFS coexposure person-days (*P* = 0.03) from 2006 to 2020.

### Coexposure distribution across different racial/ethnic groups

**Fig. 5.**
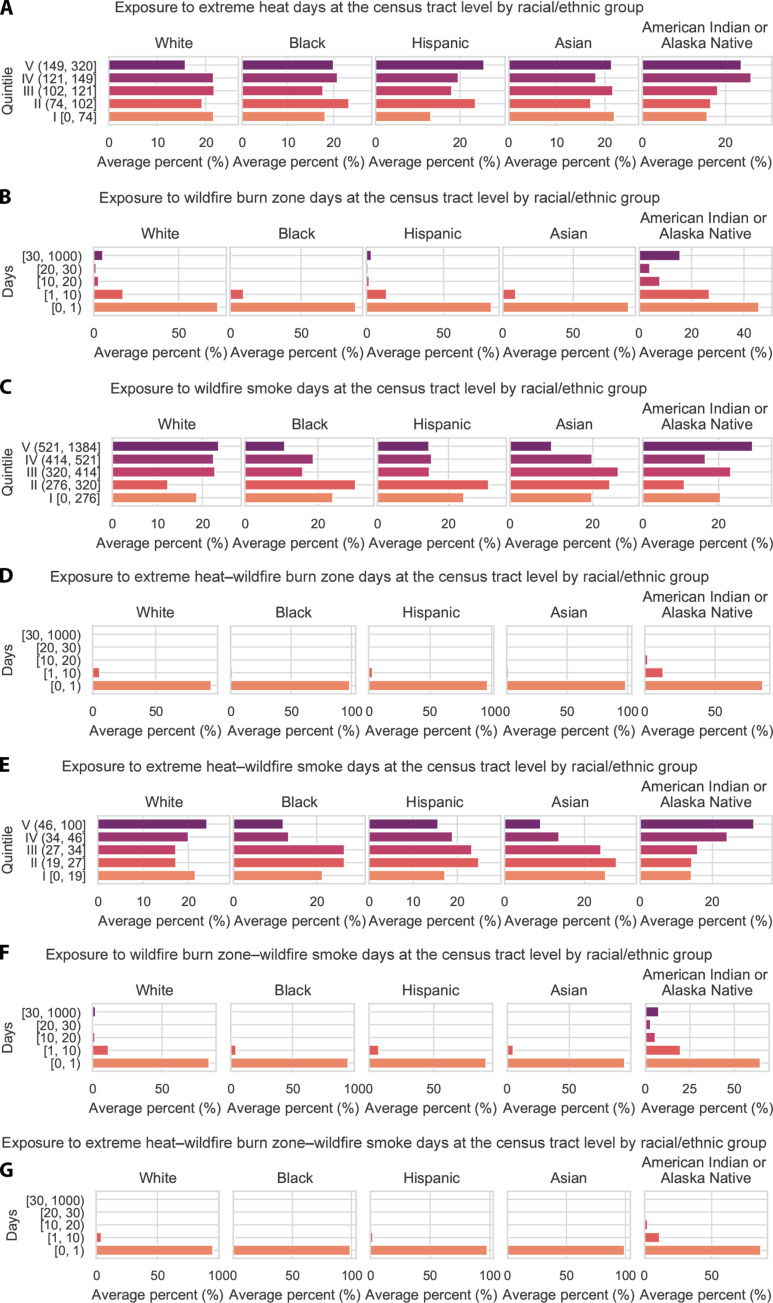
Average relative census tract–level racial/ethnic composition across 11 Western US states, 2006–2020, by level of exposure and coexposure to three climate hazards. (**A**) EH; (**B**) WFBZs; (**C**) WFS; (**D**) EH and WFBZs; (**E**) EH and WFS; (**F**) WFBZs and WFS; and (**G**) EH, WFBZ, and WFS. The exposure and coexposure spectra in (A), (C), and (E) are presented by quintile of exposure. Quintile V is the highest exposure. For WFBZs (B) and coexposure between WFBZs and other climate hazards in (D), (F), and (G), we manually created categories due to most tracts having 0 WFBZ exposure. Manual categories help address the skewed exposure distribution. Reported percentages are relative. The plots show the percentage of individuals within each racial/ethnic group in the overall exposure category. Information about census tract racial and ethnic composition was obtained from the American Community Survey (ACS), 2016–2020 (5-year) data [via US Centers for Disease Control and Prevention/Agency for Toxic Substances and Disease Registry (CDC/ATSDR) social vulnerability index (SVI) data].

Considering single hazards, Hispanic individuals and non-Hispanic American Indian and Alaska Native individuals had disproportionate EH exposure (Fig. 5A). A total of 45% of Hispanic and 50% of non-Hispanic American Indian and Alaska Native individuals lived in census tracts in the top two quintiles of EH exposure, compared to 40% or less for other groups. Non-Hispanic American Indian and Alaska Native individuals, along with non-Hispanic white individuals, were disproportionately exposed to WFBZ and WFS (Fig. 5, B and C).

For coexposures, American Indian and Alaska Native individuals faced the most EH-WFBZ (Fig. 5D), EH-WFS (Fig. 5E), WFBZ-WFS (Fig. 5F), and EH-WFBZ-WFS (Fig. 5G) coexposures. Non-Hispanic white individuals also had disproportionate exposure to wildfire-related hazards. A higher percentage of American Indian and Alaska Native and non-Hispanic white individuals experienced coexposure to EH-WFS (Fig. 5E), with 55 and 44% in the top two quintiles of EH-WFS coexposure, respectively, as opposed to 35% or less for other groups.

In a secondary analysis, we assessed differential coexposures (among tracts that experienced at least one WFBZ), comparing tracts overlapping with tribal lands versus those that did not. We found that from 2006 to 2020, tracts located on tribal lands versus those that were not had more average coexposure days per tract: 2 versus 1 EH-WFBZ days, 38 versus 35 EH-WFS days, 24 versus 10 WFBZ-WFS days, and 2 versus 1 EH-WFBZ-WFS days.

### Co-occurring climate hazards and social vulnerability

**Fig. 6.**
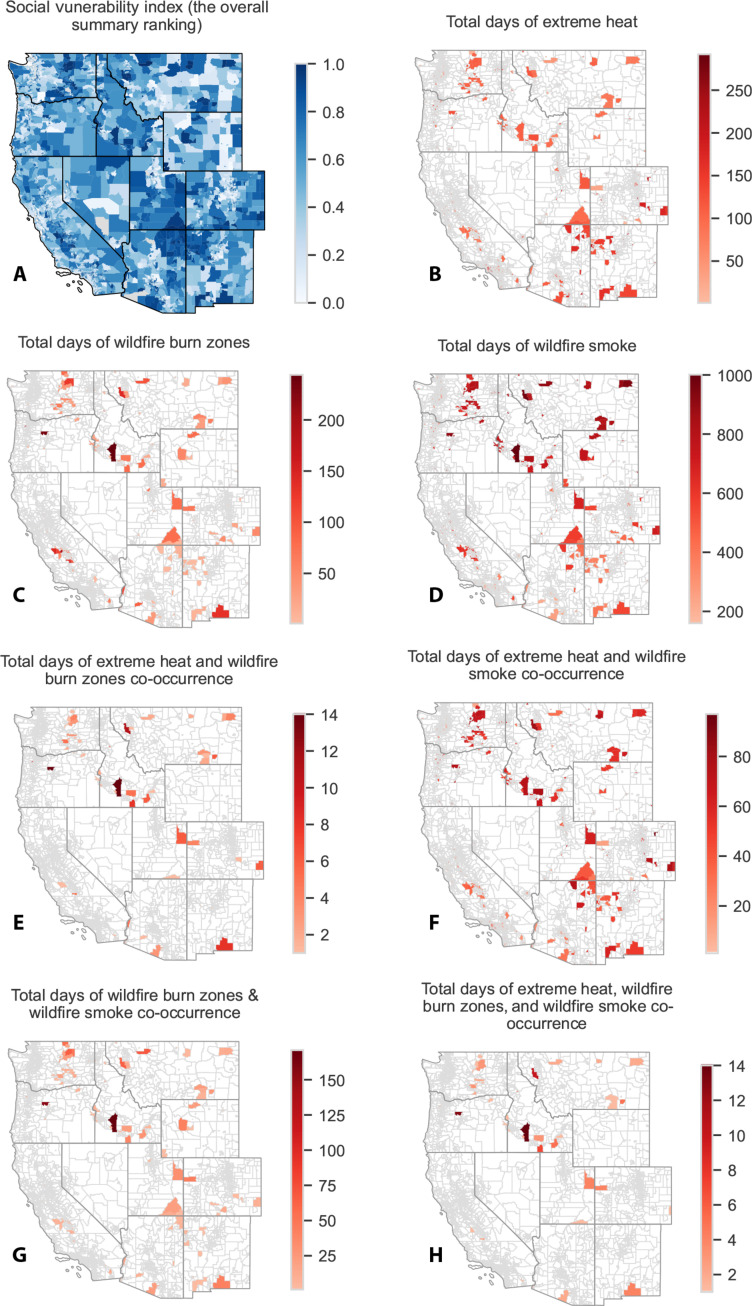
Spatial distribution of census tract–level SVI and co-occurrence of high social vulnerability with three climate hazards across 11 Western US states, 2006–2020. (**A**) SVI; (**B**) high SVI and EH; (**C**) high SVI and WFBZs; (**D**) high SVI and WFS; (**E**) high SVI and EH and WFBZs; (**F**) high SVI and EH and WFS; (**G**) high SVI and WFBZs and WFS; and (**H**) high SVI and EH, WFBZs, and WFS. High social vulnerability was defined as SVI > 0.9 or the top 10% nationwide vulnerability. The CDC produced SVI using ACS data from 2016 to 2020 (5 years).

We used the US Centers for Disease Control and Prevention (CDC)’s social vulnerability index (SVI) to identify tracts at potentially increased risk during exposure and coexposure days (Fig. 6A). Northeast Arizona, Northwest New Mexico, large portions of Utah, and parts of all other states had higher SVI (closer to 1). Segmenting to census tracts in the top 10% of the SVI (>0.9), we found that nearly every state, except for Nevada, had high vulnerability tracts that experienced single or coexposure to the three hazards. Idaho had the most coexposure to EH-WFBZ-WFS in high-SVI tracts (Fig. 6H).

### The most affected tracts and their sociodemographic characteristics

**Fig. 7.**
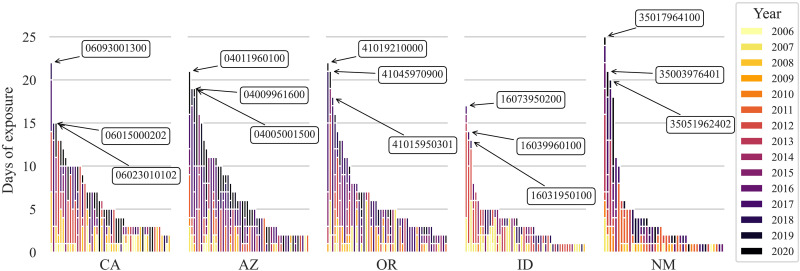
Census tracts most exposed to the co-occurrence of extreme heat, wildfire burn zone, and wildfire smoke in the five most affected states: California, Arizona, Oregon, Idaho, and New Mexico, 2006–2020. Plots are grouped by state, ranked from most to least exposed census tract, and fill colors represent the year when the coexposure occurred.

We identified five states with the most coexposure to the three hazards: California, Arizona, Oregon, Idaho, and New Mexico (Fig. 7). Within these states, specific tracts also experienced disproportionate exposure (Fig. 7). For example, a tract in New Mexico had 25 days of coexposure to EH-WFBZ-WFS during the study period.

Relative to the national or Western state average, residents of the most WFBZ-coexposed census tracts generally had sociodemographic characteristics indicating higher vulnerability (e.g., higher proportion living with a disability, of older age, or living in poverty) (Table 3). For example, 9 of the 10 most WFBZ-WFS–affected census tracts were in Idaho and California, experiencing over 300 days of cumulative coexposure compared to the Western state average of 2.4 days per census tract from 2006 to 2020. Most of these census tracts had disproportionately older, disabled, and poorer residents than the average in the Western US. Consider census tract 16049960100 in Idaho, which had 627 WFBZ-WFS coexposure days from 2006 to 2020. In this tract, 34.5% of the population was 65 or older, 28.6% had disabilities, and 22.7% lived below 150% of the poverty line. These figures are higher than the national averages of 16.8% (*29*), 12.7% (*30*), and 19.4% (*30*), respectively.

**Table 3.** Sociodemographic characteristics of the 10 census tracts with the highest cumulative climate hazard coexposure in 11 Western US states, 2006–2020. Census tract sociodemographic information was obtained from the ACS, 2016–2020 (5-year) data (via CDC/ATSDR SVI data). N/A, not applicable.

	State	Census tract number	County	Population aged 65 and older (%)	Population with disabilities (%)	Non-white race/ethnicity (%)	Population living below 150% of poverty line (%)	Tract-specific coexposure days, 2006–2020 N	11 Western US state average coexposure days per census tract, 2006–2020, N
National average*	All			16.8	12.7	42.2	19.4	N/A	N/A
11 Western US state average	AZ, CA, CO, ID, MT, NM, NV, OR, UT, WA, and WY			11.4	5.0	48.0	20.9	N/A	N/A
(A) EH and WFBZs	AZ	4007000800	Gila	31	29.4	46.5	51.7	44	0.1
	AZ	4007001300	Gila	20.5	22.8	73.7	43.7	38	
	NM	35017964100	Grant	36.2	24.4	32.4	30.2	27	
	OR	41045970900	Malheur	13.1	13.9	29.5	21	27	
	OR	41019210000	Douglas	29.7	22.6	9.1	29.5	26	
	AZ	4011960100	Greenlee	25.4	19.6	60.4	26.3	25	
	AZ	4005001500	Coconino	36.3	14.7	17.3	15.8	25	
	NV	32007951700	Elko	13.7	15.2	74.4	31.4	25	
	AZ	4009961600	Graham	8.2	12.7	53.7	18	24	
	NM	35003976401	Catron	37.3	24.3	26.7	53.9	23	
(B) EH and WFS	AZ	4011960100	Greenlee	25.4	19.6	60.4	26.3	100	31.7
	CO	8087000400	Morgan	19.9	11.5	49.3	18.6	97	
	CO	8087000500	Morgan	12.8	10.7	69.5	34.4	97	
	CO	8087000300	Morgan	16.6	11.3	42.6	18.2	95	
	CO	8087000600	Morgan	10.1	7.4	59.8	25.3	92	
	CO	8071000800	Las Animas	20	19.3	40.1	23	92	
	NM	35051962402	Sierra	39.4	19.2	42.5	34.3	90	
	NM	35017964200	Grant	35.5	21.7	25.4	26.9	89	
	CO	8063962100	Kit Carson	17.2	14	27.2	22.7	87	
	CO	8087000700	Morgan	14.1	11.8	26.4	22.1	86	
(C) WFBZs and WFS	ID	16049960100	Idaho	34.5	28.6	6.3	22.7	627	2.4
	ID	16029960200	Caribou	15.7	15.5	5.7	16	478	
	CA	6093001300	Siskiyou	26.3	21.1	38	35.7	438	
	CA	6093000800	Siskiyou	28.9	18.7	21.7	27.7	403	
	CA	6107002701	Tulare	32.4	25.7	26.6	45.9	387	
	AZ	4005001500	Coconino	36.3	14.7	17.3	15.8	378	
	ID	16085970100	Valley	47.7	29.4	6.7	24.8	340	
	ID	16035970100	Clearwater	36.6	24.4	3.8	21.4	318	
	ID	16037960200	Custer	33.2	27	14.8	27.2	311	
	ID	16079960200	Shoshone	28.8	29.7	7	29.3	308	
(D) EH, WFBZs, and WFS	NM	35017964100	Grant	36.2	24.4	32.4	30.2	25	0.12
	NV	32007951700	Elko	13.7	15.2	74.4	31.4	24	
	CA	6093001300	Siskiyou	26.3	21.1	38	35.7	22	
	OR	41019210000	Douglas	29.7	22.6	9.1	29.5	22	
	OR	41045970900	Malheur	13.1	13.9	29.5	21	21	
	NM	35003976401	Catron	37.3	24.3	26.7	53.9	21	
	AZ	4011960100	Greenlee	25.4	19.6	60.4	26.3	21	
	NM	35051962402	Sierra	39.4	19.2	42.5	34.3	20	
	AZ	4007940400	Gila	7.9	12.7	98.2	58.6	19	
	AZ	4009961600	Graham	8.2	12.7	53.7	18	19	

*National average for the population aged 65 and older (%) and the poverty rate were retrieved from data.census.gov. The national average for the Non-White race/ethnicity (%) was obtained by subtracting the White, non-Hispanic population from the total population.

Some, though not all, of the census tracts most coexposed to EH-WFBZ-WFS and EH-WFS had a higher-than-average percentage of non-white residents (Western US average = 48.0%). For example, the most EH-WFS–coexposed tract (100 cumulative days from 2006 to 2020) had a population made up of 60.4% non-white individuals. Among census tracts most exposed to all three hazards, we observed populations disproportionately consisting of people of older age, with disabilities, and living in poverty. Census tract 4007940400 in Gila County, Arizona, had 19 EH-WFBZ-WFS cumulative coexposure days (compared to the Western state average of 0.12 days) and had an exceptionally vulnerable population: 98.2% non-white individuals and 58.6% living in poverty [in comparison to the national averages (*30*–*32*)].

## DISCUSSION

Between 2006 and 2020 in 11 Western US states, we tracked three heat-related, climate-driven hazards at the census tract daily level: EH, WFBZs, and WFS PM_2.5_ exposure. We found frequent exposure to single hazards. On average, each census tract experienced 32 days of exposure to at least one hazard per year. Coexposures occurred less frequently and exhibited substantial spatial variation. The EH-WFS was the most common coexposure, with tracts experiencing an average of 2 days per year. WFBZ and WFS exposures showed distinct spatial patterns, with WFBZ exposure more confined and WFS more dispersed with the highest prevalence in Northern California, Oregon, Eastern Washington, Idaho, Montana, and Wyoming. EH, WFS, and EH-WFS exposures all increased in prevalence over the study period, with regional differences. Increasing EH exposure occurred in California, Colorado, and Nevada, and increasing WFS exposure took place in Arizona, Colorado, Oregon, and Washington. Only California and New Mexico experienced increasing EH-WFS coexposure tract-days, and just Arizona and New Mexico saw increasing EH-WFBZ-WFS coexposure tract-days over the study period. We observed American Indian and Alaska Native individuals disproportionately faced all coexposures. We also highlighted census tracts with dual-high coexposures and social vulnerability.

Our finding of substantial spatiotemporal variability of single and coexposure to EH, WFBZ, and WFS aligns with existing literature using single-state data (*24*, *25*). In a county-level Washington State analysis from 2006 to 2018, Austin *et al.* (*24*) found that July to September had the highest prevalence of coexposure to heat and ambient PM_2.5_ during the wildfire season. We also found that census-tract level EH-WFS exposure peaked during summer. Rosenthal *et al.* (*25*) used 3-km resolution data in 2020 and found that 16.5 million people in California were affected at least once by EH-WFS. Our work evaluated this coexposure in 11 Western states over 15 years, uncovering 2.4-billion cumulative person-days of EH-WFS exposure. While we found an increasing trend in EH-WFS tract-days over time in the Western US, in line with prior evidence (*26*), WFBZ tract-days did not increase in any state, and EH-WFBZ-WFS tract-days only increased in Arizona and New Mexico from 2006 to 2020.

Our research findings have public health implications. In addition to increased morbidity and mortality in human populations tied to EH and wildfires separately (*13*, *15*, *33*, *34*), researchers have reported synergistic effects on health. For example, Shaposhnikov *et al.* (*7*) found a multiplicative effect of air pollution from wildfires during the 44-day 2010 Moscow heat wave on mortality. Chen *et al.* (*27*) showed synergistic effects of EH-WFS on cardiorespiratory hospitalizations in California from 2006 to 2019. They identified stronger effects in more disadvantaged communities, highlighting the importance of identifying disadvantaged communities that also experience disproportionate exposure, as we did with SVI. We also found that the census tracts experiencing the most EH-WFBZ, WFBZ-WFS, and EH-WFBZ-WFS coexposures simultaneously tended to have a higher proportion of older adults and people living in poverty. Public health practitioners and policymakers should consider coexposure disparities and differential responses when considering targeted interventions and follow-up health studies.

A body of environmental justice literature documents disproportionate EH exposure among communities of color in the US (*35*, *36*). Reports of inequitable WFS exposure are mixed (*28*, *37*–*39*), and no studies have evaluated WFBZ exposure by race/ethnicity. Only Rosenthal *et al.* (*25*) assessed coexposure disparities, studying EH-WFS. They found that in 2020, Hispanic and Black Californians were less likely, and white Californians were more likely than expected to experience EH-WFS coexposures (*25*). They attributed this to the stochastic nature of WFS exposure and more white individuals living in rural areas closer to WFBZ (*25*). In descriptive analyses, we identified disproportionate coexposure to EH-WFS, WFBZ-WFS, and EH-WFBZ-WFS among non-Hispanic white and American Indian and Alaska Native individuals in 11 Western states. This finding aligns with reports of elevated long-term WFS exposure in California among non-Hispanic white and American Indian and Alaska Native populations from 2006 to 2020 (*37*). We found evidence that census tracts overlapping with tribal lands had higher WFBZ exposure than other nonoverlapping tracts. American Indian people may have higher WFBZ exposure because they tend to live in rural areas with higher wildfire risk (*40*). Cultural burning practices used by tribes to support food, health, and cultural traditions can also improve ecosystem health and reduce the chances of catastrophic wildfires (*41*). Our use of satellite imagery could have classified some of these cultural burns as WFBZs, potentially elevating the estimated exposure among American Indian people. However, tribes have faced liability for burns, potentially restricting their use (*42*). A California bill went into effect in 2022, exempting cultural burns from having a state-approved burn plan and removing liability risk for those practicing cultural burning (*43*). As climate change accelerates, exposure patterns may shift, and populations may migrate (*44*, *45*), necessitating ongoing environmental justice studies. Any observed disparities may be underestimated because of heightened health-protective behaviors (*46*) and resources among advantaged populations, such as air conditioning, filtration, and higher housing quality.

Our research had limitations. In typical climatological studies, a 30-year period establishes a baseline for defining an EH day. In contrast, our methodology used a 5-year period, which may better handle increasing temperatures over time and human adaptation. Our analysis defined coexposure based on simultaneous climate hazards co-occurring in the same tract-day rather than the overlapping duration of climate events. While we operationalized coexposure in day units, climate hazards often span multiple days, and multiday exposures likely have worse health impacts than single-day exposures. Future studies should evaluate alternatives to our simplified coexposure definition, for example, considering the duration of coexposure in analysis. In addition, to estimate person-days of exposure, we made the simplifying assumption that everyone in the tract experienced the same exposure. To identify WFBZs, we relied on archived MODIS C6.1 MCD41A1 active fire/hot spot data to capture wildfire occurrence (*47*). These data do not distinguish between prescribed burns and wildfires, and thus, our analysis inherently includes both.

Given the small sample size of 15 years in trend analysis, small *P* values reported should be treated as indicative of a possible trend. For the states with *P* values near 0.05 or larger, more years of data are required to conclude the lack of a trend (*48*).

Further, while several new remote sensing products have improved resolution and sensitivity regarding fire radiative power (e.g., LANDSAT 30 m OLI 8/9 with data available since 2022), MODIS performs adequately in most settings. Therefore, we used the MODIS product based on its general comparability with newer products, its long archive (November 2000 to present), and its computationally tractable resolution of 1 km. We considered the Monitoring Trends in Burn Severity (MTBS) database (*49*), which provides detailed WFBZ maps. However, MTBS only maps fires larger than 1000 acres in the Western US, missing smaller wildfires. We also evaluated wildfire datasets from the National Interagency Fire Center (NIFC) (*50*), which integrates local-, state-, and national-level reporting of wildland fire incidents. Both MODIS and NIFC data have strengths and limitations (*51*). NIFC data can have multiple start and end dates due to inconsistencies across agencies’ reporting, with some dates unknown. Because accurate dates are critical for creating coexposure days, we preferred MODIS. Nevertheless, MODIS wildfire detection is limited by fire size and environmental conditions (*47*, *51*). Recent studies have shown that MCD64A1 C6, the version we used, has improved burned area mapping accuracy compared to previous versions (*52*). In addition, MODIS products do not provide information about fire size or differentiate between prescribed fires and wildfires. Future studies could link MODIS data or other satellite imagery with existing wildfire boundary databases, such as MTBS, to isolate wildfires from prescribed burns or to evaluate coexposures from medium and large wildfires. We are also interested in adopting clustering and other machine learning methods for pinpointing coexposure hot spots in future work.

While we used daily census tract–level data across 11 states, improving the spatial scale and coverage of previous studies, this granularity also presented challenges. Notably, states contain varying numbers of census tracts because the number is based on population size. For example, California has more than 8000 tracts compared to fewer than 500 in Idaho or Montana. To improve interpretability, we presented results in total counts and per-tract averages. Further, the three selected hazards do not conform to administrative census tract boundaries. WFBZs readily traverse multiple tracts within a day.

WFBZ exposure also tends to occur in rural and suburban areas, so most urban areas had no WFBZ coexposure. This means that the sociodemographic characteristics of those experiencing the WFBZ coexposures are those of rural and suburban dwellers. Future work may wish to use spatial resolutions derived from the hazards’ inherent characteristics. Further, while our current scope was limited to the Western US, the widening spread of EH and WFS necessitates expanding these analyses to the entire US.

This study evaluated 2006–2020 coexposure to three heat-related, climate-driven hazards—EH, WFBZ, and WFS—across census tracts in 11 Western US states. We found that the average tract experienced 32 days per year of exposure to at least one hazard, increasing trends for EH and WFS (individually and EH-WFS together) but not for WFBZ and its coexposures over time for the entire 11-state area. States exhibited distinct trends in their EH-WFBZ-WFS coexposures. We also documented disparities in exposure by sociodemographic groups to prioritize mitigation, adaptation, and health intervention efforts. Identifying the location and year of co-occurring climate hazards will pave the way for future impact studies.

## MATERIALS AND METHODS

### Study design

We conducted an analysis at the census tract level in 11 Western US states from 2006 to 2020. The US Census Bureau delineates census tracts as subcounty units containing approximately 4000 people (range: 1200 to 8000). The study included all census tracts in Arizona (*n* = 1765), Colorado (*n* = 1447), Idaho (*n* = 456), Montana (*n* = 319), Nevada (*n* = 779), New Mexico (*n* = 612), Utah (*n* = 716), and Wyoming (*n* = 160). It included most census tracts in California (*n* = 9095 of 9129), Oregon (*n* = 991 of 1001), and Washington (*n* = 1766 of 1784). The 62 omitted tracts had missing sociodemographic data, consisted entirely of water, or did not intersect with exposure datasets (fig. S6).

### Datasets

#### Weather data

For daily temperature data from 2006 to 2020, we used the Regional Approaches to Climate Change gridMET maximum air temperature data (*53*). gridMET had no missingness and provided a high-resolution (^1^/_24_th degree, ~4 km^2^) gridded temperature surface for the contiguous United States. We aggregated data to the census tract level.

#### Wildfire data

We used archived MODIS C6.1 MCD41A1 active fire/hot spot data to capture wildfire occurrence (*47*). The MODIS active fire product relies on 1-km daily resolution satellite imagery to delineate pixels (yes/no) as containing an active fire each day by placing a fire point at the pixel centroid (*54*). We processed these data using ArcGIS Pro by conducting a spatial join of fire points to census tracts (using the Spatial Join tool) and calculating summary statistics by day by census tract (using the Summary Statistics as Table tool). Summary statistics provided a count of the number of fire points in each census tract daily.

#### WFS data

We used a previously developed WFS PM_2.5_ dataset from Childs *et al.* (*28*). The daily WFS PM_2.5_ concentrations were predicted using a machine learning model, with gradient-boosted trees for the architecture and the root mean square error as the loss function. The model estimated daily PM_2.5_ concentrations due to wildfires using a combination of ground, satellite, and reanalysis data sources. The model was validated using fivefold nested spatial cross-validation, with an additional layer of hyperparameter tuning using fourfold cross-validation. These estimates were derived from 2006 to 2020 in a 10-km^2^ nationwide grid. We aggregated the data into census tracts (*28*).

#### Census data and SVI

The CDC created the SVI as a comprehensive tool to gauge a community’s vulnerability to adverse health consequences of disasters (*55*). In addition to the SVI metric, the dataset provided socioeconomic and census information. The SVI has four components derived from the 5-year 2016–2020 American Community Survey (ACS). The first component considers socioeconomic factors, such as poverty and unemployment rates, per capita income, and education levels. The second evaluates household composition and disability, considering factors such as the proportion of older adults and underage persons (i.e., percentages of persons aged ≥ 65 or ≤ 17 years), the population with disabilities, and single-parent families with children under 18 years. The third component considers racial and ethnic composition and language proficiency, including the proportions of Hispanic, non-Hispanic Black, American Indian and Alaska Native, Asian, Native Hawaiian or other Pacific Islander, or other groups and individuals with English language linguistic isolation. The fourth component looks at housing conditions and transportation, incorporating factors such as housing density, availability of private vehicles, and individuals in institutionalized group quarters. Each component is ranked on a percentile scale and combined to produce the overall SVI score. This combined score is converted into a percentile rank, with values between 0 and 1, where higher values imply a greater vulnerability to natural disasters. Tracts with zero estimates for the total population (*n =* 62) were removed during the ranking process. After ranking, these tracts were added back to the SVI database but labeled as missing (*55*).

#### Shapefiles

We used a 2020 census tract shapefile and the population size from the 2016–2020 ACS [via CDC/Agency for Toxic Substances and Disease Registry (ATSDR) SVI data]. We also identified tribal lands using the US Census Bureau’s Topologically Integrated Geographic Encoding and Referencing (TIGER)/Line shapefiles representing American Indian/Alaska Native/Native Hawaiian (AIANNH) areas based on data from the 2019 census (*56*). The AIANNH shapefile contained a unique polygon for each American Indian reservation or off-reservation trust land, American Indian statistical geographic entity, Hawaiian homeland, and Alaska Native village statistical area (fig. S7). Census tract–level exposure and covariate data were combined using spatial and nonspatial joins (based on census tract ID). The final dataset consisted of 18,106 census tracts.

### Defining EH, WFBZs, WFS, heat, and coexposure days

We defined an EH day when the census tract’s daily ambient maximum temperature equaled or exceeded both a relative and an absolute threshold. For the relative threshold, we used the 95th percentile of the local maximum daily temperatures of all days during the warm season (May to September) through 2006–2010 (*57*, *58*). For the absolute threshold, we used a daily maximum temperature of ≥90°F (32.22°C) (*59*, *60*). The relative threshold considers a community’s local climate, infrastructure, and acclimatization of the population. Using both absolute and relative thresholds is important for defining EH, preventing the use of unreasonably low-temperature thresholds that might arise from relying solely on relative thresholds. In our study, 28.5% (*n* = 5163) of census tracts had a relative threshold of <32.22°C, and 2.7% of census tracts had a relative threshold of <25°C (*n* = 486) (fig. S8).

A WFBZ tract-day occurred when the MODIS active fire dataset reported at least one fire point present in a tract on a given day. The MODIS active fire product routinely detects fires in an area of 1000 m^2^ but does not distinguish between wildfires and prescribed burns (*54*, *61*).

A WFS PM_2.5_ tract-day occurred when the modeled WFS PM_2.5_ concentration in a tract (*28*) exceeded 0 μg/m^3^ for a given day. As a sensitivity analysis, we also considered days where WFS PM_2.5_ concentration exceeded 5 μg/m^3^. Throughout the article, we used WFS to denote the modeled PM_2.5_ specific to wildfire emissions.

A coexposure day was defined by two or more events occurring simultaneously in the same census tract on the same day. For a sensitivity analysis, we defined a 2-day coexposure day, when two events happened on the same day or adjacent days (within a 2-day window). For instance, if EH happened a day before a WFBZ in a census tract, then we considered it a 2-day coexposure day.

We used the nonparametric Mann-Kendall test to evaluate trends over time in the number of individual or co-occurring climate-driven hazards in the 11 Western states combined and by state. The statistical analysis was performed using Python package pyMannKendall 1.4.3 (*62*).

### Defining exposure to climate hazards

We used the following metrics to describe the risk of single climate hazards and co-occurring climate hazards for each census tract *i.*

### Total exposure tract-days per year

$$T_{\text{year}}=\frac{\sum_{y=2006}^{2020}E_{y}}{N_{\text{years}}}$$

where $E_{y}$ denotes the tract-specific exposure days in year y; $\sum_{y=2006}^{2020}E_{y}$ represents the sum of exposure days from 2006 to 2020; $N_{\text{years}}=15$, the number of years; and $T_{\text{year}}$ is the total exposure or coexposure tract-days per year.

### Total exposure days per census tract per year

$$T_{\text{ct},\text{year}}=\frac{1}{N_{\text{cts}}}\frac{\sum_{i=1}^{N_{\text{cts}}}E_{i}}{N_{\text{years}}}$$

where $N_{\text{cts}}=18,106$, the number of census tracts in the 11 Western states; $\sum_{i=1}^{N_{\text{cts}}}E_{i}$ represents the sum of exposure days for all census tracts from 2006 to 2020; $N_{\text{years}}=15$, the number of years; and $T_{\text{ct},\text{year}}$ is the total exposure days per census tract per year.

### Total person-day exposure

$$\text{PD}E_{i}=P_{i}\times E_{i}$$

where $P_{i}$ represents the population size of census tract *i*; $E_{i}$ represents the exposure days for census tract *i* from 2006 to 2020; and $\text{PD}E_{i}$ represents the person-day exposure for census tract *i* from 2006 to 2020

$$T_{\text{person},\text{year}}=\frac{\sum_{i=1}^{N_{\text{cts}}}{\text{PDE}}_{i}}{N_{\text{years}}}$$

where $\sum_{i=1}^{N_{\text{cts}}}{\text{PDE}}_{i}$ represents the sum of person-days of exposure for all census tracts from 2006 to 2020; $N_{\text{years}}=15$, the number of years; and $T_{\text{person},\text{year}}$ is the total exposure person-days per year.

### Total exposure person-days per census tract per year

$$T_{\text{person},\text{ct},\text{year}}=\frac{1}{N_{\text{cts}}}\frac{\sum_{i=1}^{N_{\text{cts}}}\text{PD}E_{i}}{N_{\text{years}}}$$

where $N_{\text{cts}}=18,106$, the number of census tracts; $\sum_{i=1}^{N_{\text{cts}}}\text{PD}E_{i}$ represents the sum of person-days of exposure for all census tracts from 2006 to 2020; $N_{\text{years}}=15,$ the number of years; and $T_{\text{person},\text{ct},\text{year}}$ is the total exposure person-days per census tract per year.

### Total exposure tract-days per year for a state

$$T_{S,\text{year}}=\frac{\sum_{i=1}^{N_{\text{cts},S}}E_{i}}{N_{\text{years}}}$$

where $N_{\text{cts},S}$ represents the number of census tracts in state S; $\sum_{i=1}^{N_{\text{cts},S}}E_{i}$ represents the sum of exposure days for all tracts in state S from 2006 to 2020; $N_{\text{years}}=15,$ the number of years; and $T_{S,\text{year}}$ is the total exposure tract-days per year for state S.

### Total exposure person-days per year for a state

$$T_{\text{person},S,\text{year}}=\frac{\sum_{i=1}^{N_{\text{cts},S}}\text{PD}E_{i}}{N_{\text{years}}}$$

where $\sum_{i=1}^{N_{\text{cts},S}}\text{PD}E_{i}$ represents the sum of person-days of exposure for all census tracts in state S from 2006 to 2020; $N_{\text{years}}=15$, the number of years; and $T_{\text{person},S,\text{year}}$ represents the total exposure person-days for the state per year. In the above definitions, the number of exposure days was replaced by the number of coexposure days to calculate coexposures to climate hazards.

We examined exposure to the three hazards from 2006 to 2020 in relation to relative census tract–level racial/ethnic composition, including Hispanic, non-Hispanic white, non-Hispanic Black, non-Hispanic Asian, and non-Hispanic American Indian and Alaska Native persons. Because the proportion of people in each racial/ethnic category differed markedly (e.g., in the study area, 49.9% of people identified as non-Hispanic white and 1.1% identified as non-Hispanic American Indian and Alaska Native), we first computed overall exposure quintiles for EH and WFS hazards and then presented the percentage of each racial/ethnic group that fell into each quintile. Because of the high frequency of tract-days with zero WFBZ exposure, categories based on quintiles were less informative. Therefore, we manually created categories for WFBZ and their coexposure with other climate hazards. If exposures were evenly distributed across a racial/ethnic group, then 20% of individuals from that group would appear in each quintile of exposure.

In a secondary analysis, we completed a spatial join of the census tract shapefile and the tribal land shapefile to identify tracts overlapping with tribal lands. We then further restricted the analysis to tracts that experienced at least one WFBZ during the study period and compared the distribution of coexposures within tribal and nontribal land tracts to determine whether disproportionate exposure occurred on tracts overlapping with tribal lands.
