# Supplementary material for: Coexposure to extreme heat, wildfire burn zones, and wildfire smoke in the Western US from 2006 to 2020
Source: Sci Adv. 2025 Apr 30;11(18):eadq6453. doi: 10.1126/sciadv.adq6453 (PMC12042893; doi:10.1126/sciadv.adq6453)
Supplement: Supplementary file 1 — Figs. S1 to S8 Tables S1 to S4 [file sciadv.adq6453_sm.pdf]

Science Advances
AAAS

Supplementary Materials for

Coexposure to extreme heat, wildfire burn zones, and wildfire smoke in the Western US from 2006 to 2020

Jie K. Hu *et al.*

Corresponding author: Jie K. Hu, hu.3125@osu.edu; Ana Trišović, ana_tris@mit.edu

Sci. Adv. **11**, eadq6453 (2025)
DOI: 10.1126/sciadv.adq6453

This PDF file includes:

Figs. S1 to S8
Tables S1 to S4

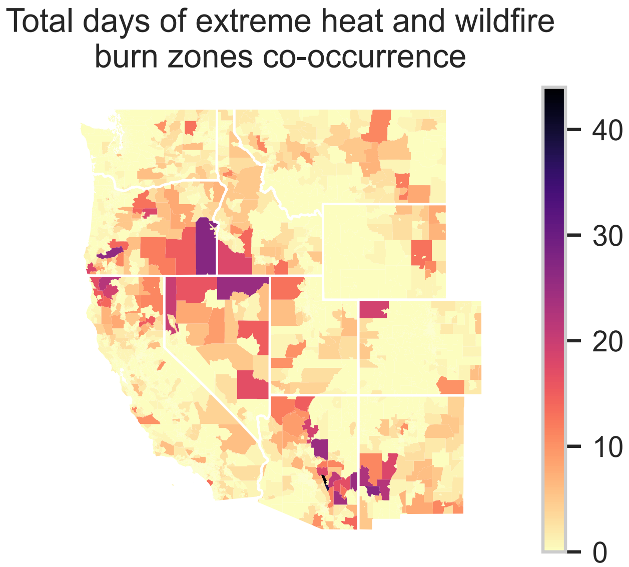

Fig. S1.

Spatial pattern of the total days of co-occurring extreme heat[a] and wildfire burn zone[b] exposure in 11 Western US states, 2006-2020.

Black indicates the most exposure-days and light yellow the fewest.

[a] Defined based on the local maximum daily temperature equaling or exceeding the warm season 95th percentile and 90°F.

[b] Defined as an active fire/ hot spot identified by MODIS C6.1 MCD41A1 in the census tract.

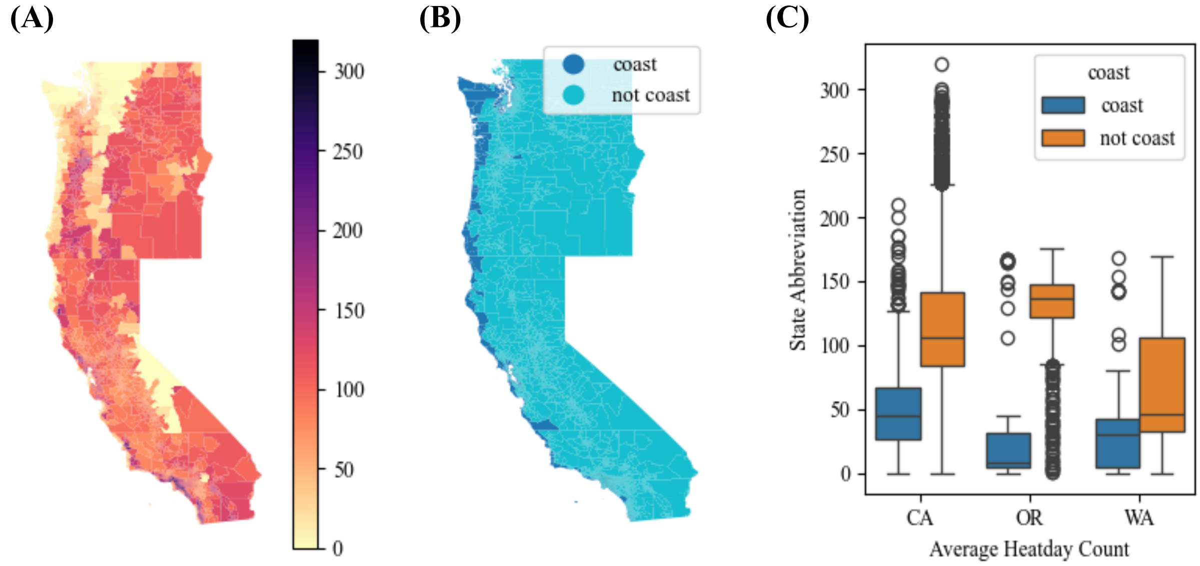

Fig. S2.
Extreme heat tract-days by coast vs. not-coast on the West Coast, 2006-2020

As the California coastline appeared to have higher extreme heat exposure than non-coastline areas (A), we categorized the census tracts in the western coastline states as “coast” and “not coast” (B). On average, there is no significant difference in the number of high-heat days between coastal and non-coastal census tracts (C).

(A)

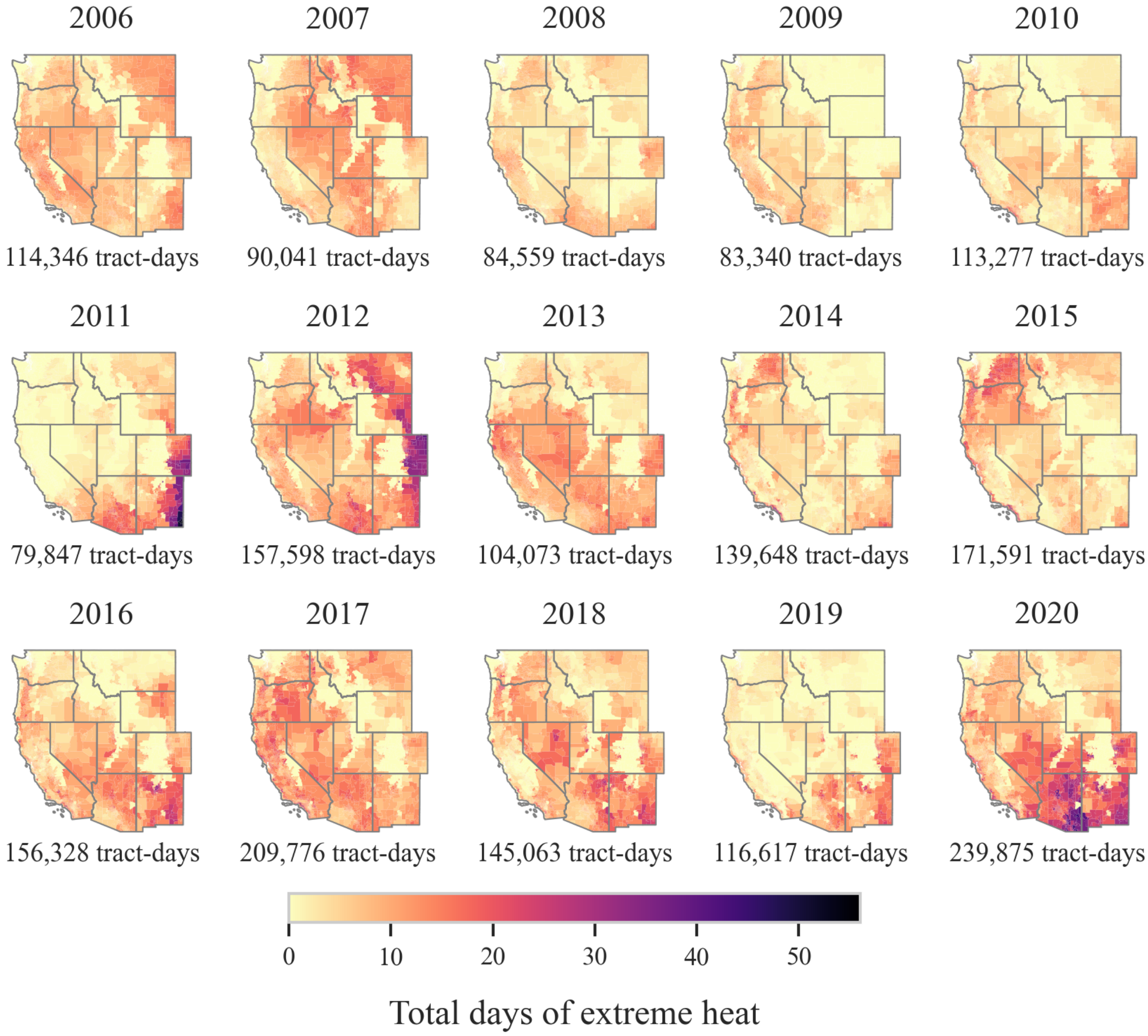
2006
114,346 tract-days
2007
90,041 tract-days
2008
84,559 tract-days
2009
83,340 tract-days
2010
113,277 tract-days
2011
79,847 tract-days
2012
157,598 tract-days
2013
104,073 tract-days
2014
139,648 tract-days
2015
171,591 tract-days
2016
156,328 tract-days
2017
209,776 tract-days
2018
145,063 tract-days
2019
116,617 tract-days
2020
239,875 tract-days
0
10
20
30
40
50
Total days of extreme heat

(B)

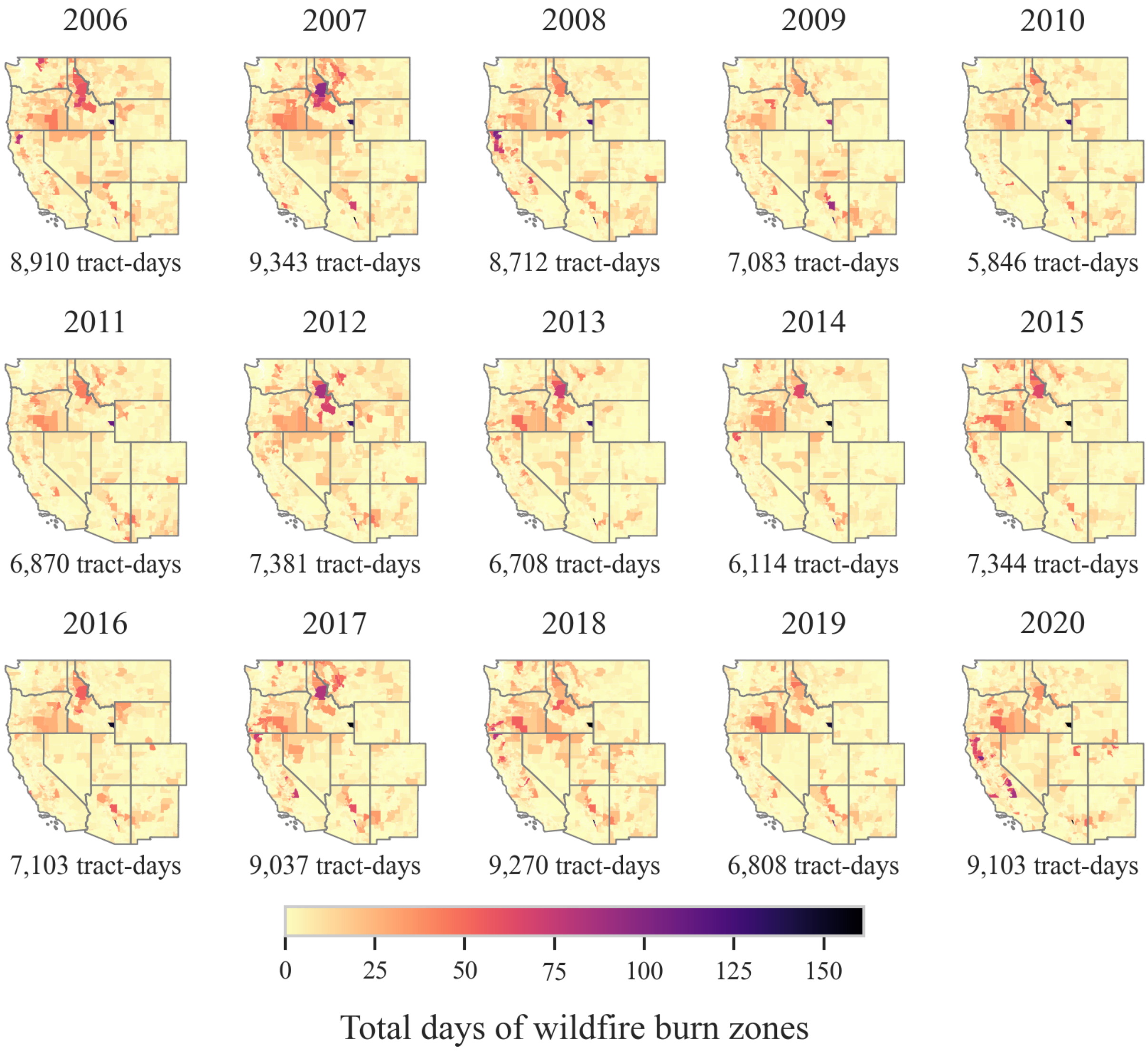
2006
8,910 tract-days
2007
9,343 tract-days
2008
8,712 tract-days
2009
7,083 tract-days
2010
5,846 tract-days
2011
6,870 tract-days
2012
7,381 tract-days
2013
6,708 tract-days
2014
6,114 tract-days
2015
7,344 tract-days
2016
7,103 tract-days
2017
9,037 tract-days
2018
9,270 tract-days
2019
6,808 tract-days
2020
9,103 tract-days
0
25
50
75
100
125
150
Total days of wildfire burn zones

(C)

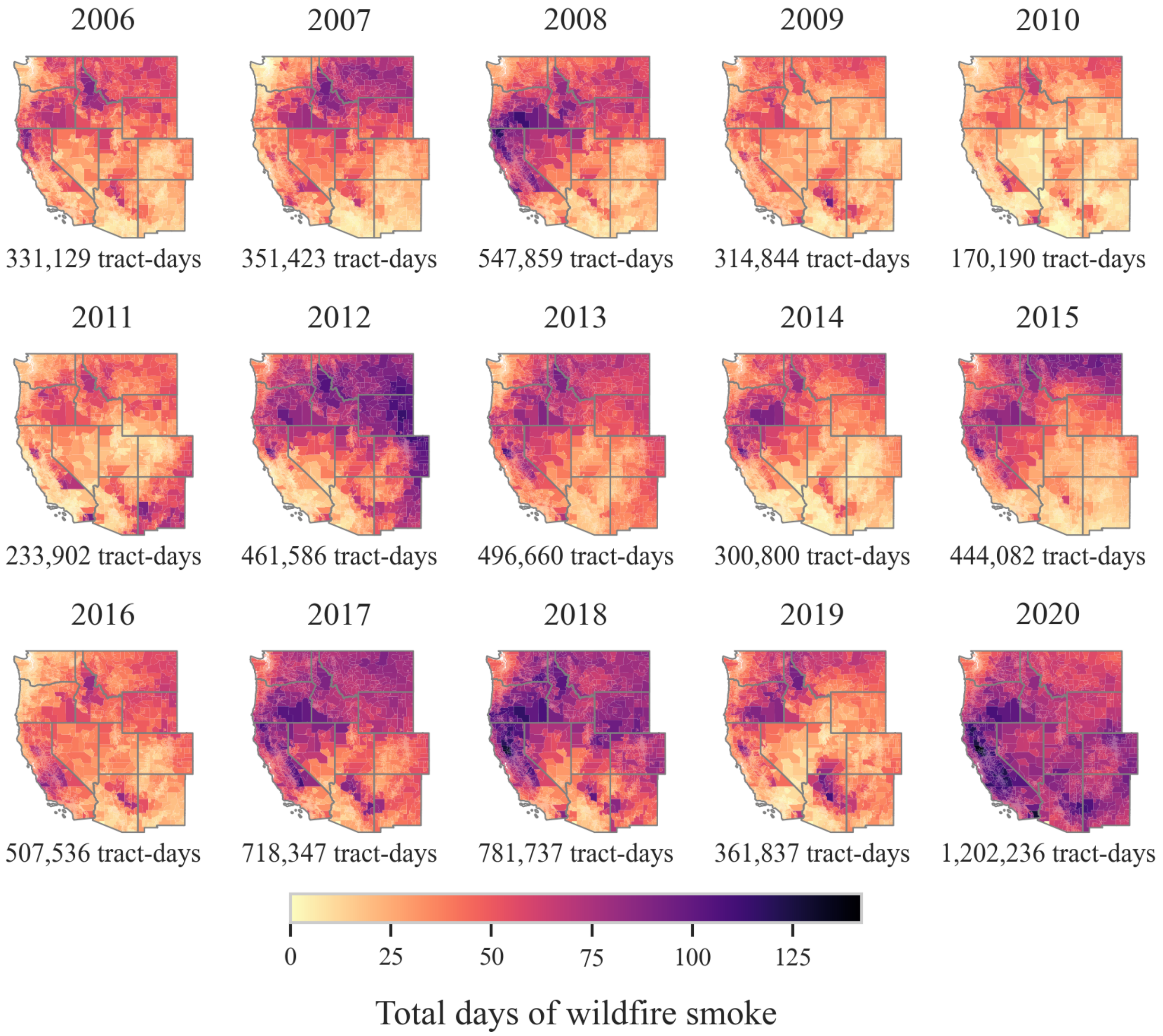
2006
331,129 tract-days
2007
351,423 tract-days
2008
547,859 tract-days
2009
314,844 tract-days
2010
170,190 tract-days
2011
233,902 tract-days
2012
461,586 tract-days
2013
496,660 tract-days
2014
300,800 tract-days
2015
444,082 tract-days
2016
507,536 tract-days
2017
718,347 tract-days
2018
781,737 tract-days
2019
361,837 tract-days
2020
1,202,236 tract-days
0
25
50
75
100
125
Total days of wildfire smoke

(D)

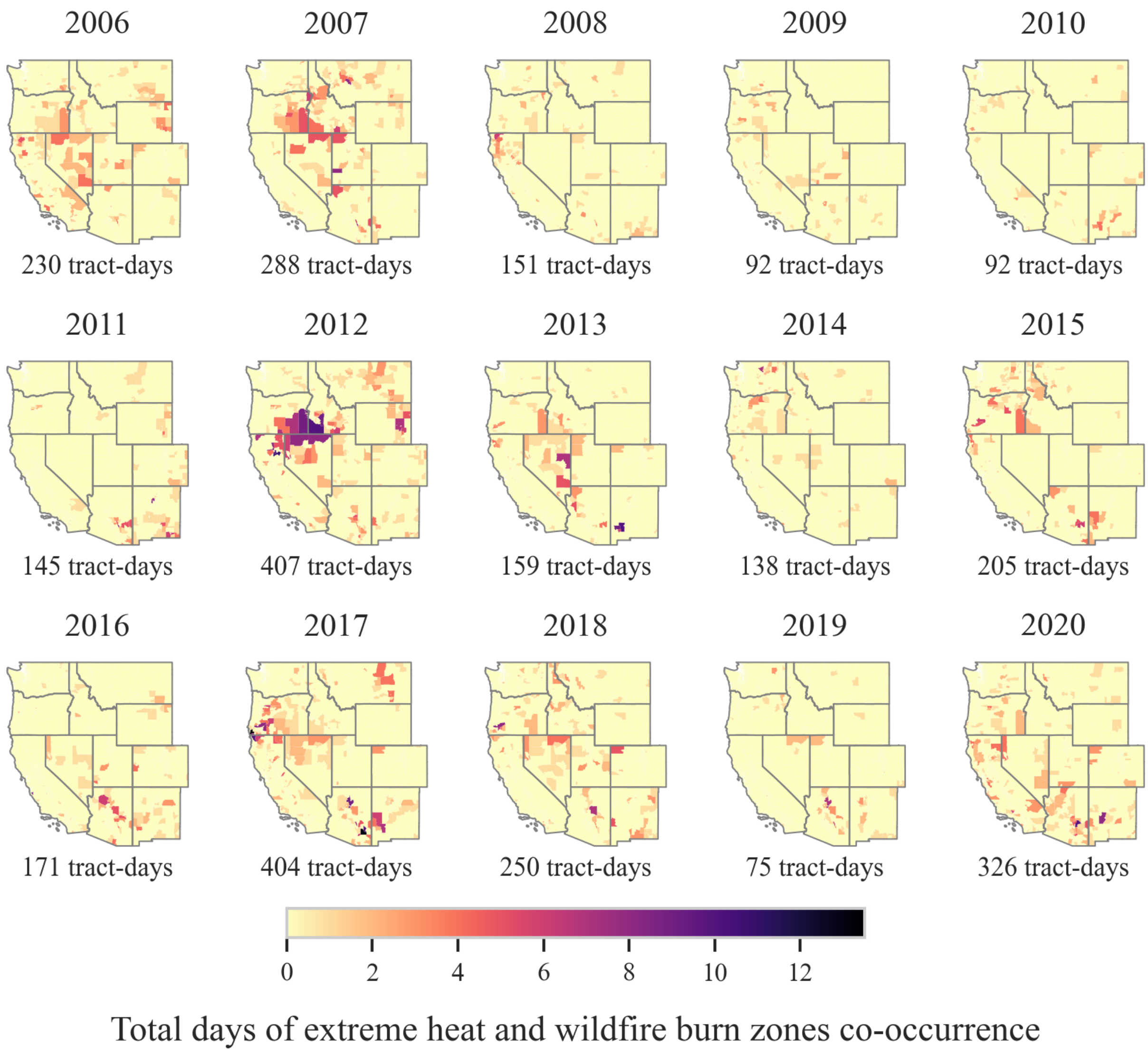
2006
230 tract-days
2007
288 tract-days
2008
151 tract-days
2009
92 tract-days
2010
92 tract-days
2011
145 tract-days
2012
407 tract-days
2013
159 tract-days
2014
138 tract-days
2015
205 tract-days
2016
171 tract-days
2017
404 tract-days
2018
250 tract-days
2019
75 tract-days
2020
326 tract-days
0
2
4
6
8
10
12
Total days of extreme heat and wildfire burn zones co-occurrence

(E)

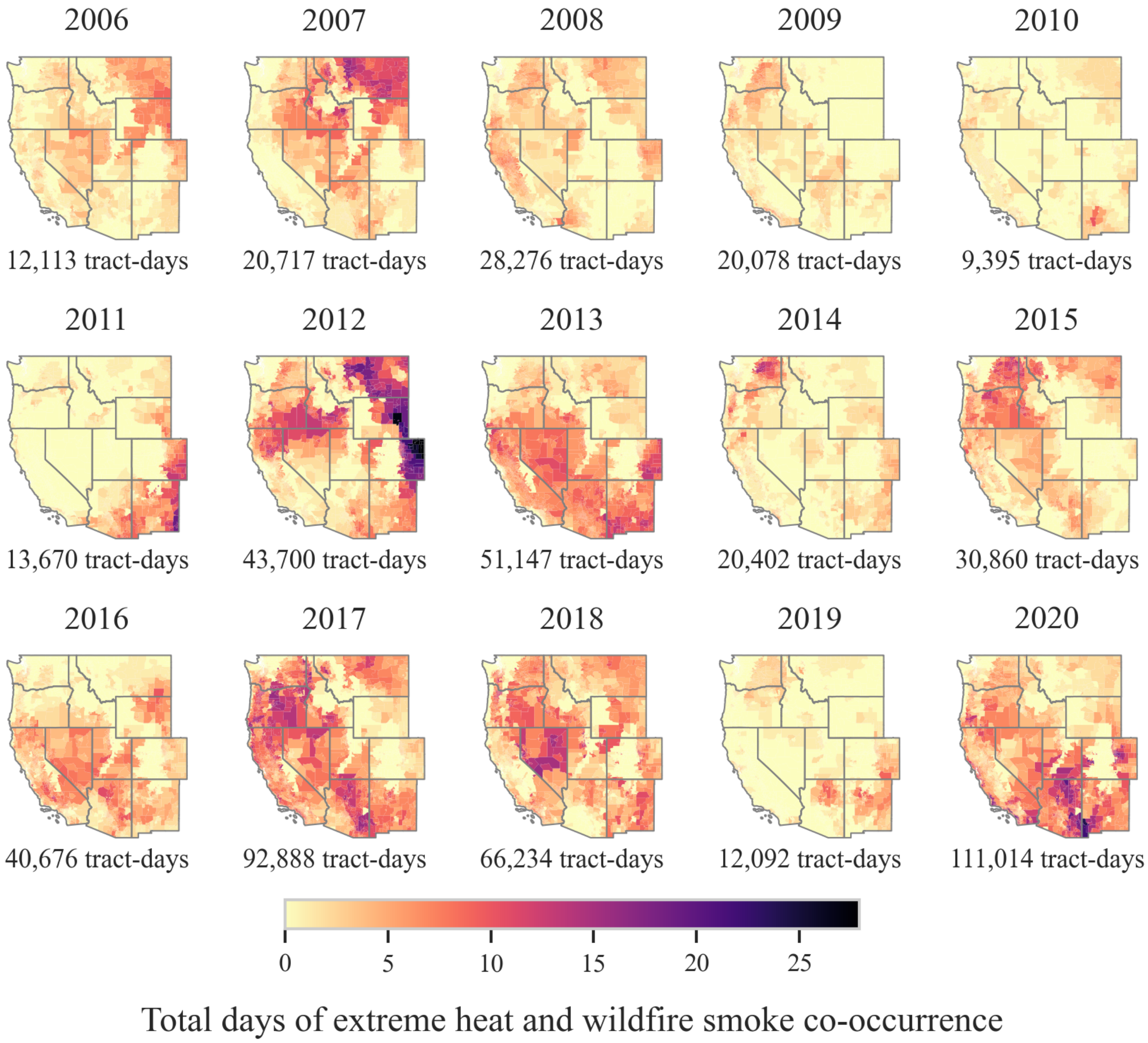
2006
2007
2008
2009
2010
12,113 tract-days
20,717 tract-days
28,276 tract-days
20,078 tract-days
9,395 tract-days
2011
2012
2013
2014
2015
13,670 tract-days
43,700 tract-days
51,147 tract-days
20,402 tract-days
30,860 tract-days
2016
2017
2018
2019
2020
40,676 tract-days
92,888 tract-days
66,234 tract-days
12,092 tract-days
111,014 tract-days
0
5
10
15
20
25
Total days of extreme heat and wildfire smoke co-occurrence

(F)

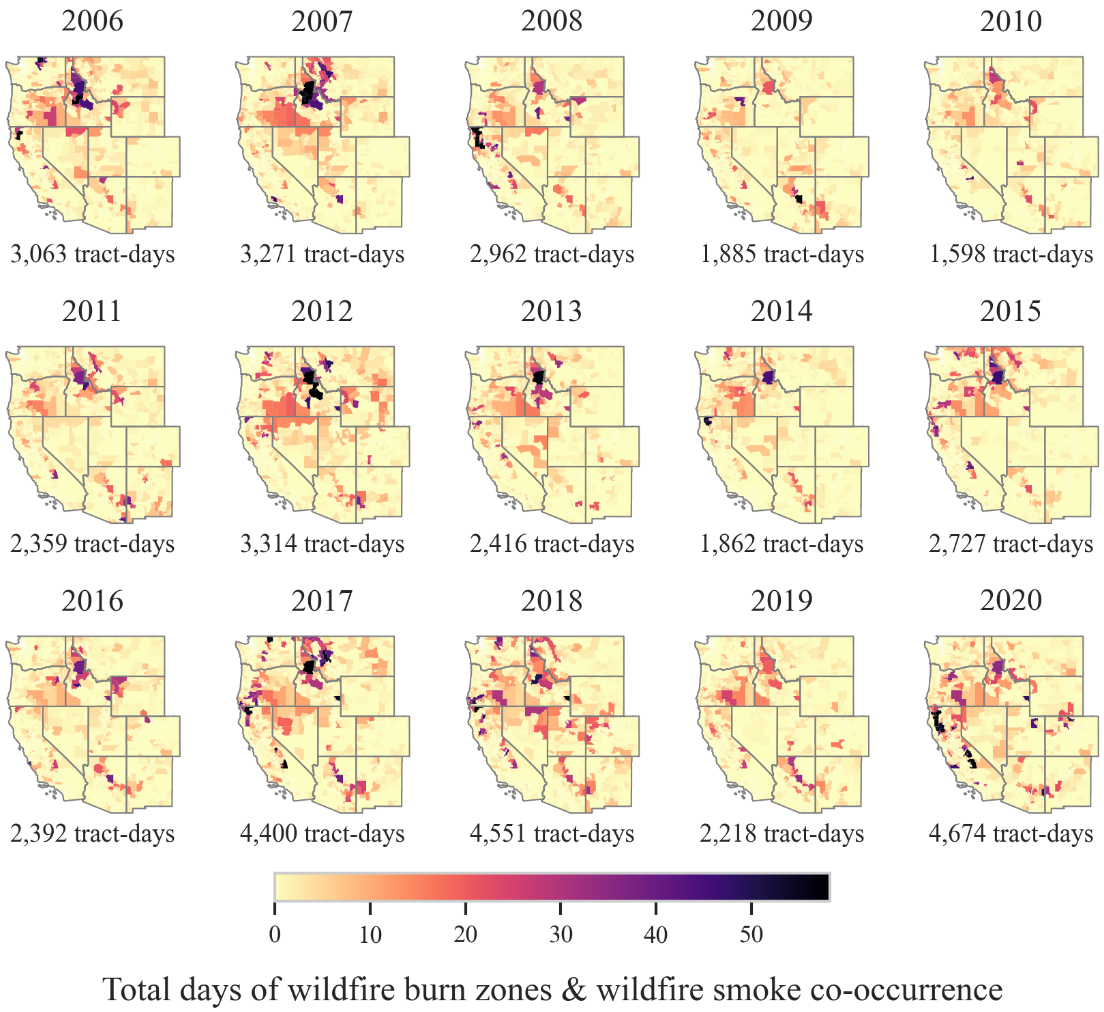
2006
3,063 tract-days
2007
3,271 tract-days
2008
2,962 tract-days
2009
1,885 tract-days
2010
1,598 tract-days
2011
2,359 tract-days
2012
3,314 tract-days
2013
2,416 tract-days
2014
1,862 tract-days
2015
2,727 tract-days
2016
2,392 tract-days
2017
4,400 tract-days
2018
4,551 tract-days
2019
2,218 tract-days
2020
4,674 tract-days
0
10
20
30
40
50
Total days of wildfire burn zones & wildfire smoke co-occurrence

(G)

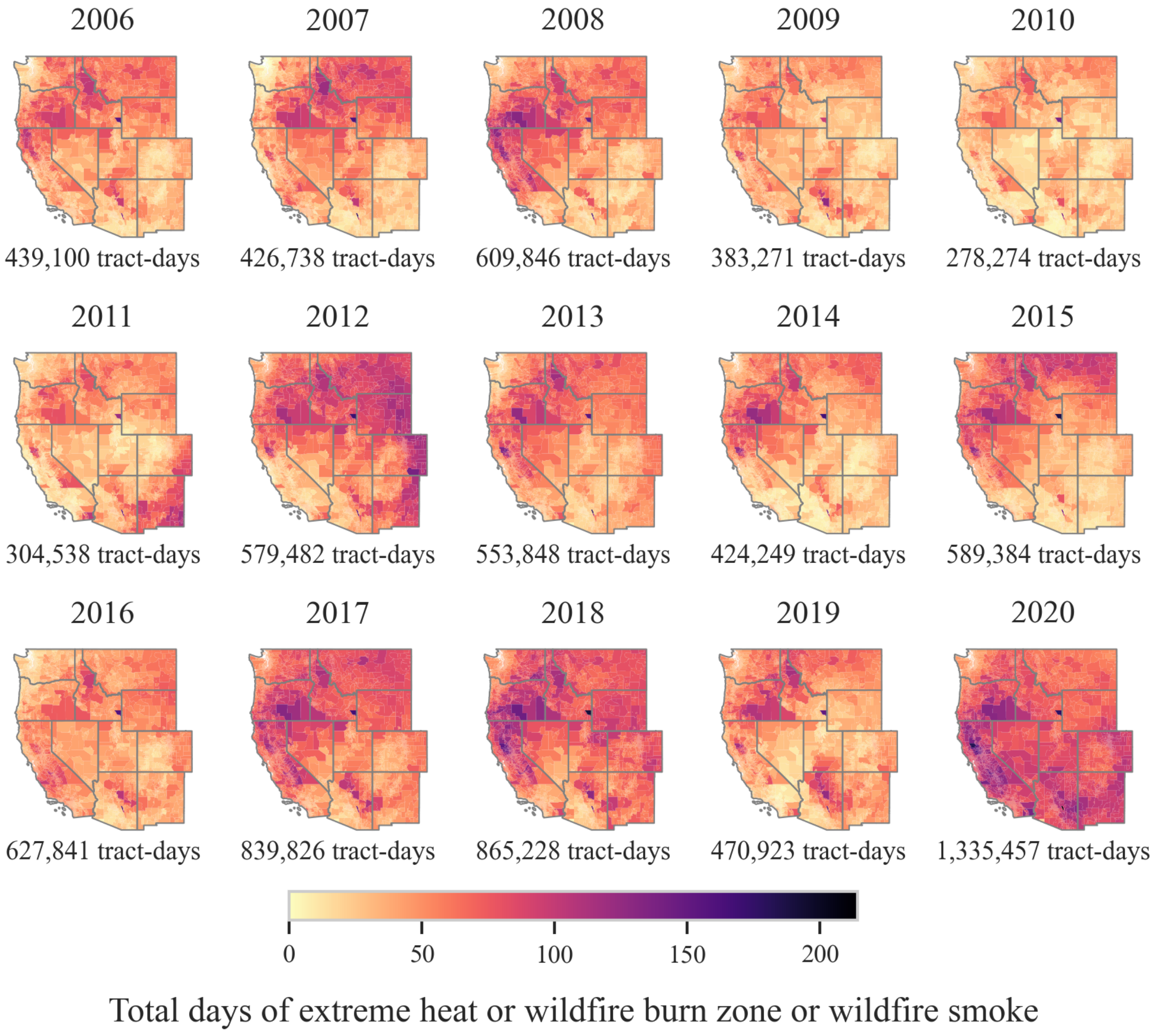
2006
439,100 tract-days
2007
426,738 tract-days
2008
609,846 tract-days
2009
383,271 tract-days
2010
278,274 tract-days
2011
304,538 tract-days
2012
579,482 tract-days
2013
553,848 tract-days
2014
424,249 tract-days
2015
589,384 tract-days
2016
627,841 tract-days
2017
839,826 tract-days
2018
865,228 tract-days
2019
470,923 tract-days
2020
1,335,457 tract-days
0
50
100
150
200
Total days of extreme heat or wildfire burn zone or wildfire smoke

(H)

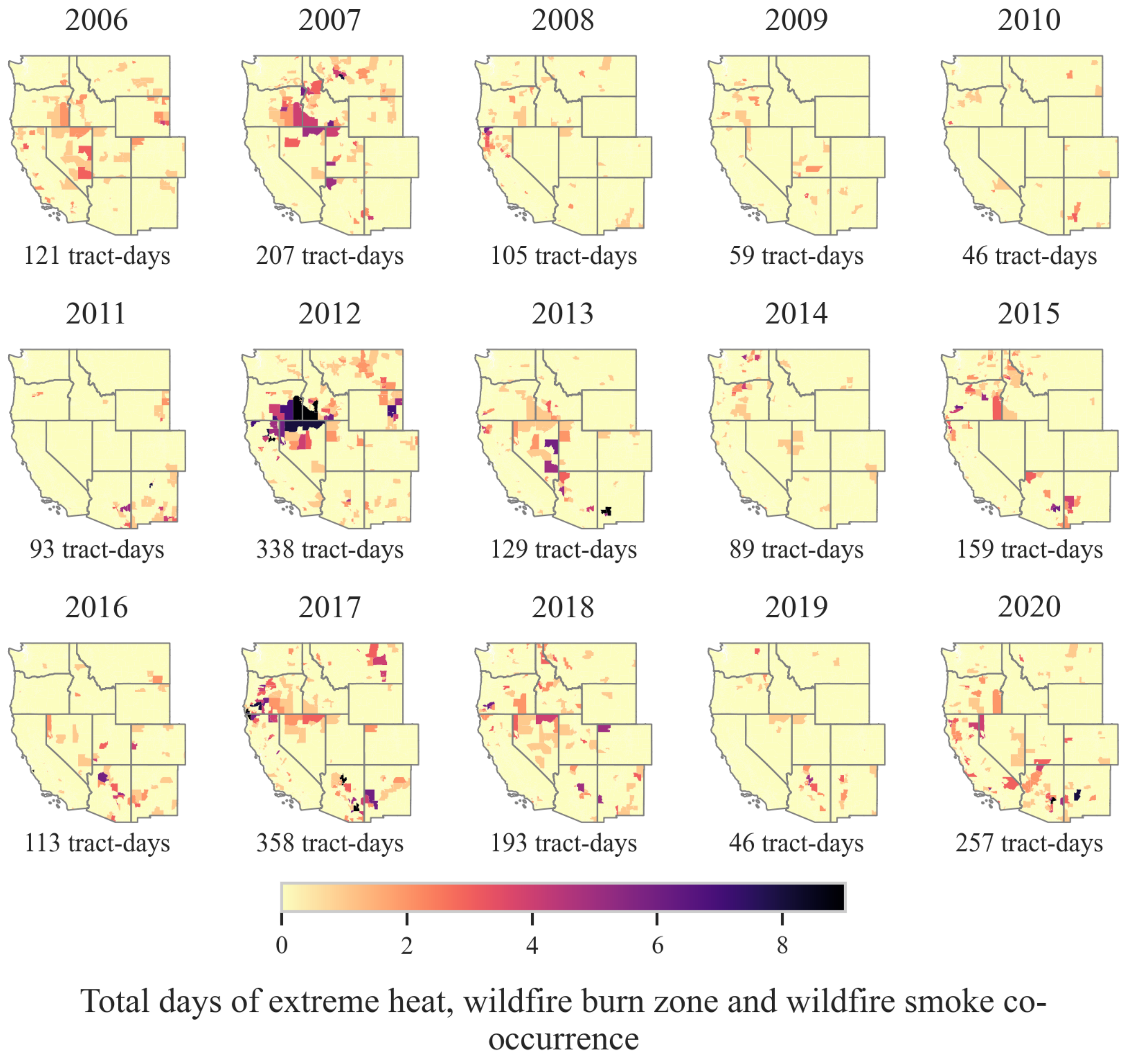

Fig. S3.

Spatial distribution of exposure to the three individual climate hazards and co-exposure to the hazards at the census tract-level in 11 Western US States, 2006-2020.
Black indicates the most exposure-days and light yellow the fewest. *P* value for Mann-Kendall test for trend in tract-days of exposure from 2006–2020 is indicated in the paratheses after each individual or co-exposure below.

(A) Extreme heat (*p*=0.02)
(B) Wildfire burn zone (*p*=1.00)
(C) Wildfire smoke (*p*=0.03)
(D) Extreme heat and wildfire burn zone (*p*=0.59)
(E) Extreme heat and wildfire smoke (*p*=0.03)
(F) Wildfire burn zone and wildfire smoke (p=0.37)
(G) Extreme heat, wildfire burn zone, *or* wildfire smoke (*p*=0.02)
(H) Extreme heat, wildfire burn zone, *and* wildfire smoke (*p*=0.46)

(A)

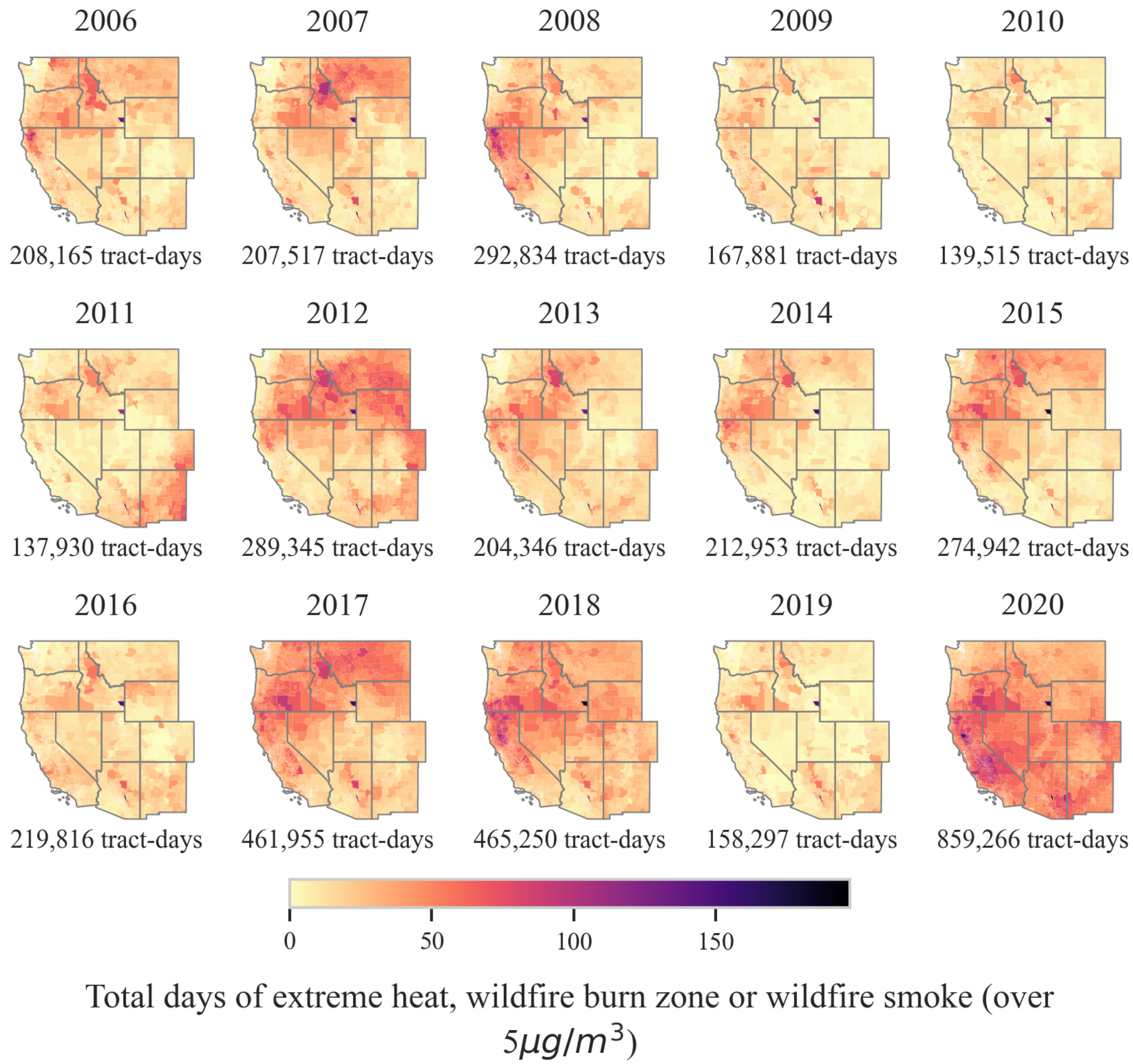
2006
208,165 tract-days
2007
207,517 tract-days
2008
292,834 tract-days
2009
167,881 tract-days
2010
139,515 tract-days
2011
137,930 tract-days
2012
289,345 tract-days
2013
204,346 tract-days
2014
212,953 tract-days
2015
274,942 tract-days
2016
219,816 tract-days
2017
461,955 tract-days
2018
465,250 tract-days
2019
158,297 tract-days
2020
859,266 tract-days
0
50
100
150
Total days of extreme heat, wildfire burn zone or wildfire smoke (over $5\mu g/m^3$)

(B)

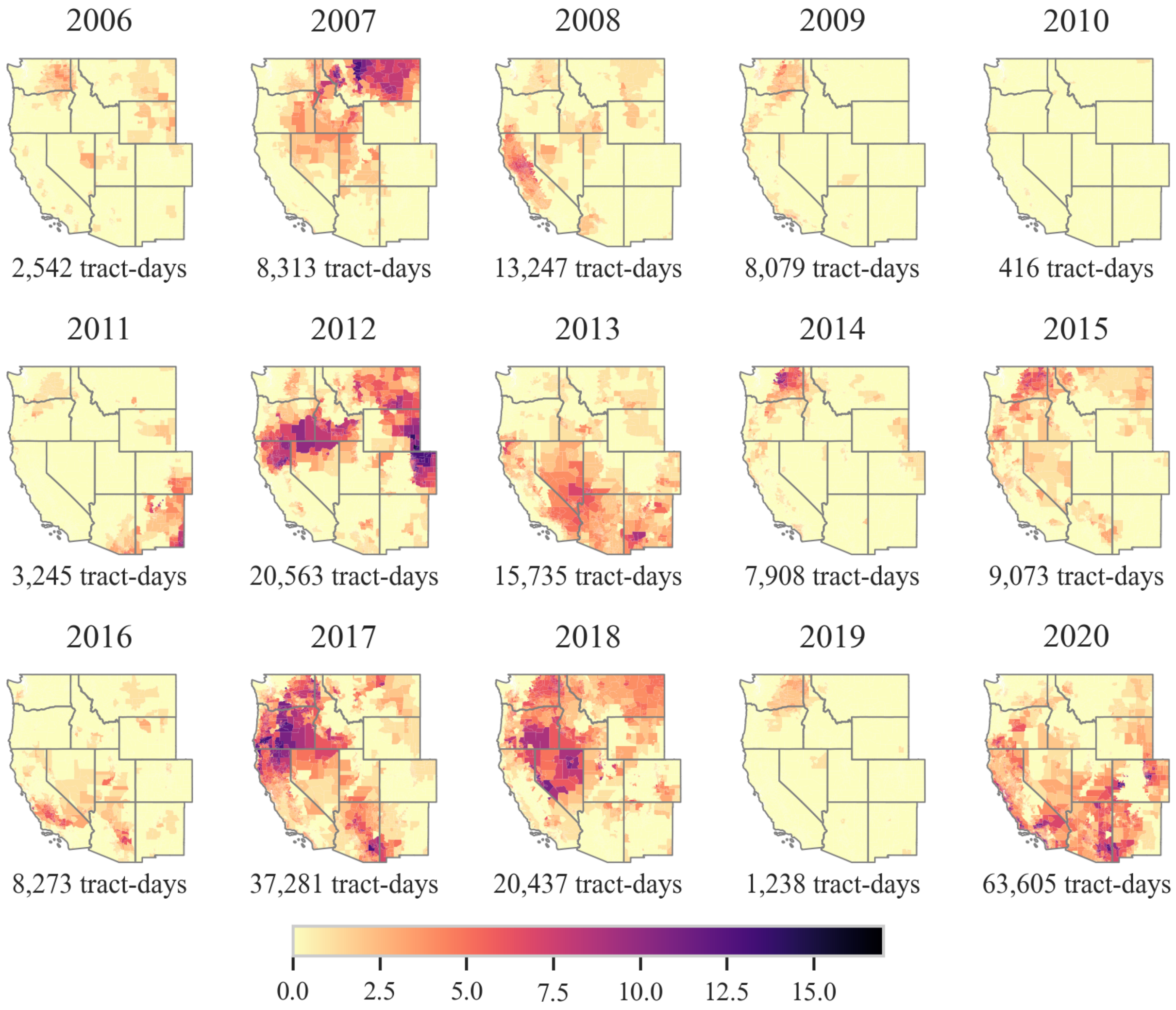

Total days of extreme heat and wildfire smoke (over $5\mu g/m^3$) co-occurrence

(C)

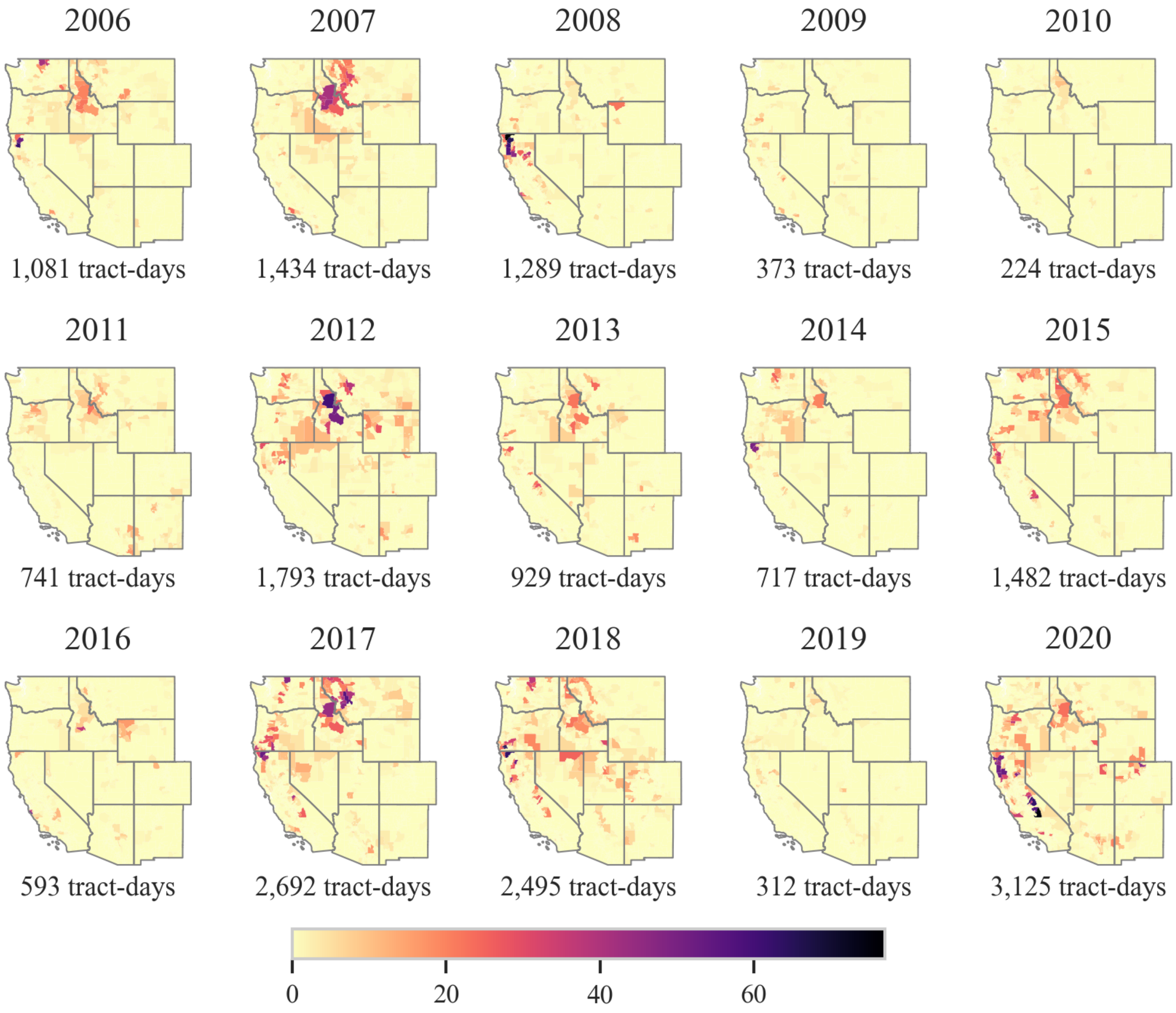

Total days of wildfire burn zone and wildfire smoke (over $5\mu g/m^3$) co-occurrence

(D)

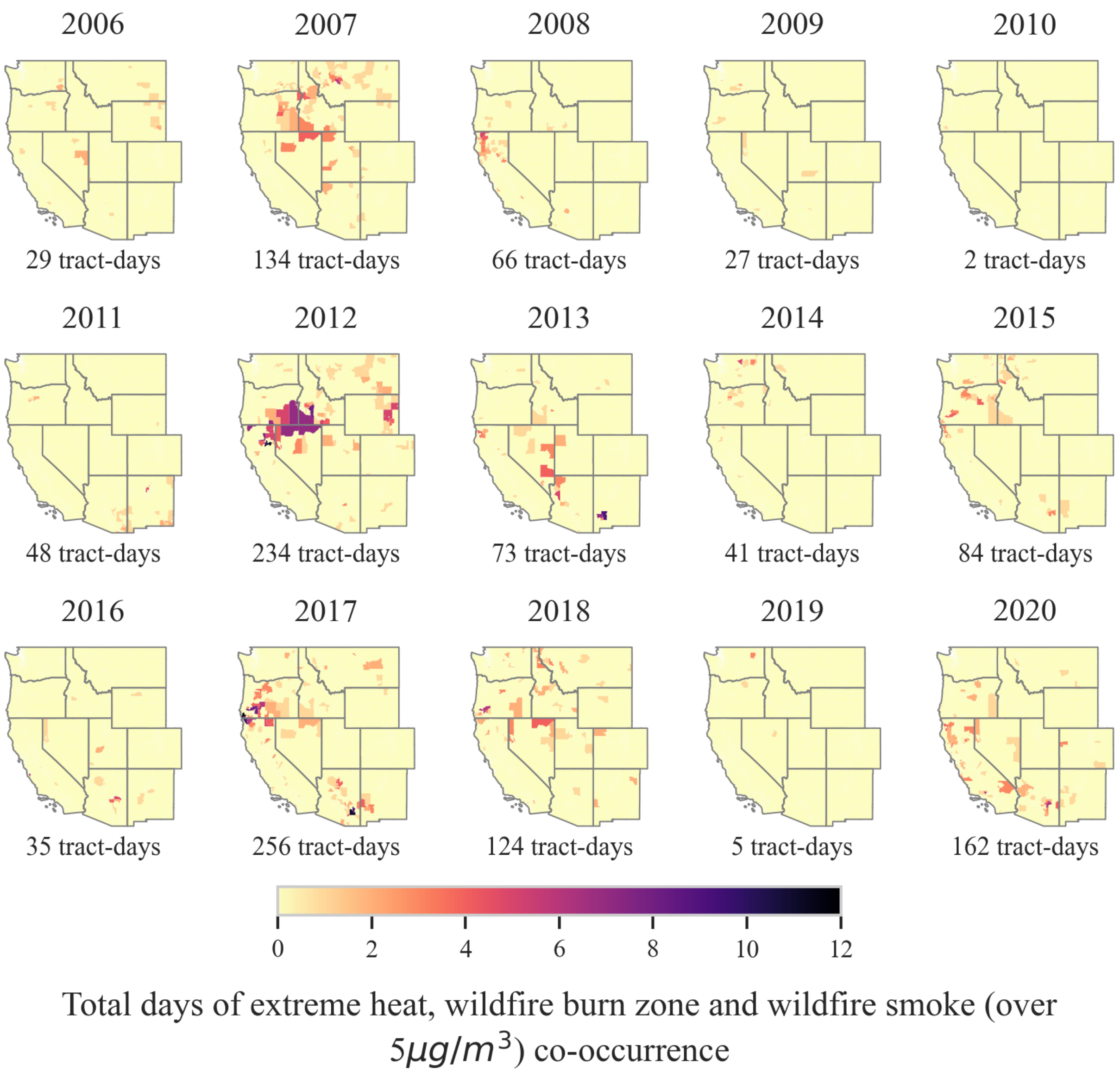

Fig. S4.

Spatial distribution of exposure to the three climate hazards at the census tract-level in 11 Western US States, 2006-2020.

Figure 1 updated to show wildfire smoke days when wildfire $PM_{2.5}$ >5μg/m^3 rather than >0μg/m^3. Black indicates the most exposure-days and light yellow the fewest.

(A) Total co-exposure tract-days to extreme heat, wildfire burn zones, or wildfire smoke
(B) Total tract-days of extreme heat and wildfire smoke co-exposure
(C) Total tract-days of wildfire burn zone and wildfire smoke co-exposure
(D) Total tract-days of extreme heat, wildfire burn zone, and wildfire smoke co-exposure

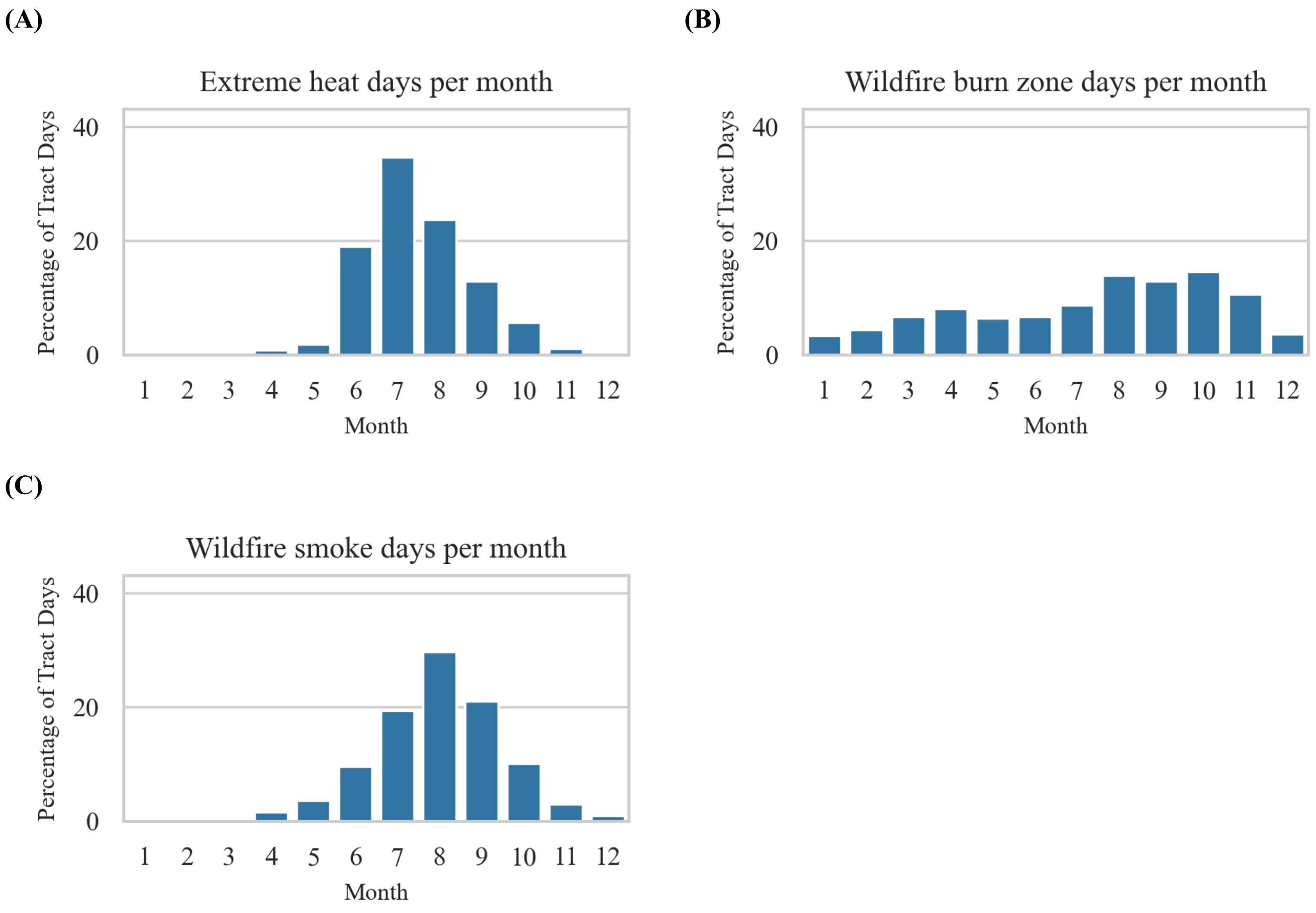

Fig. S5.

Distribution by month of census tract-days of exposure in 11 Western US States, 2006-2020.

Percentages are calculated out of total tract-days where the exposure of interest occurred.

(A) Extreme heat (defined based on the local maximum daily temperature equaling or exceeding the warm season 95th percentile and 90°F).

(B) Wildfire burn zones (defined as an active fire/ hot spot identified by MODIS C6.1 MCD41A1 in the census tract).

(C) Wildfire smoke (defined as wildfire $PM_{2.5}$ concentration over 0 $\mu g/m^3$).

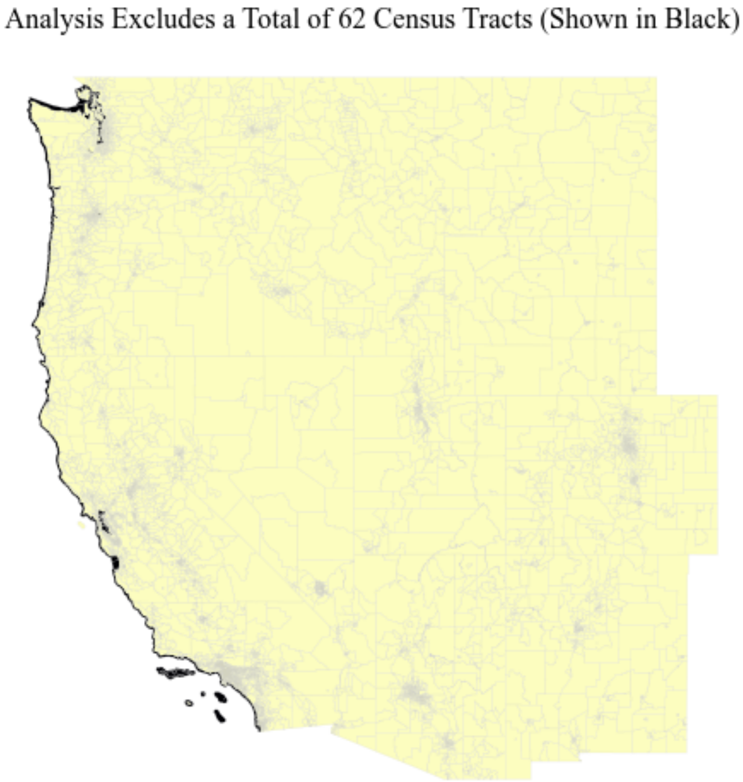

Fig. S6.
Spatial distribution of census tracts omitted from analyses in the 11 Western US states.
We excluded 62 tracts that were missing sociodemographic data or did not intersect with exposure datasets.

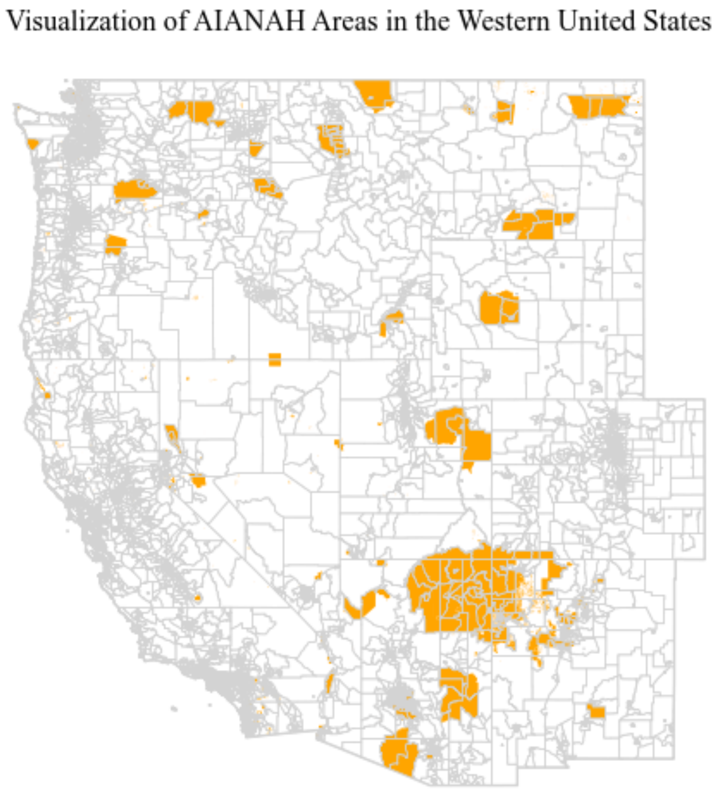

Fig. S7.
Spatial distribution of American Indian/Alaska Native/Native Hawaiian (AIANNH) areas based on data from the 2019 U.S. Census Bureau's TIGER/Line shapefiles in the 11 Western US states. The AIANAH shapefile contained a unique polygon for each American Indian reservation or off-reservation trust land, American Indian statistical geographic entity, Hawaiian Home Land, and Alaska Native Village statistical area *(57)*.

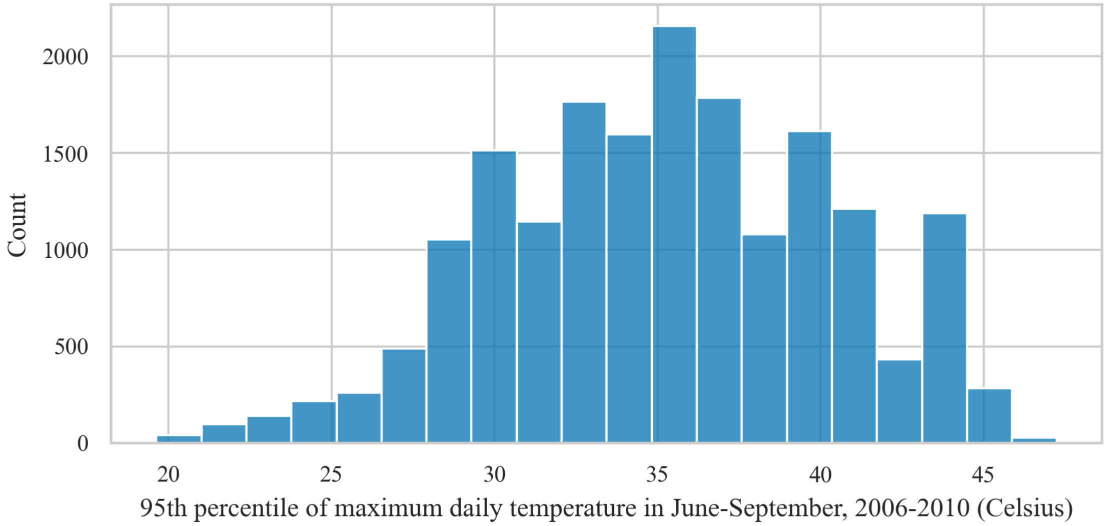

Fig. S8.
Distribution of 95th percentile of maximum daily temperature per census tract in the 11 Western US states from 2006-2010.

Table S1.
Annual average prevalence of single and co-occurring census tract-level climate hazards in 11 Western US states, 2006-2020

	**Census tract-day exposures**		**Person-day exposures**	
	**Annual average sum of tract-days of exposure**	**Annual average tract-days of exposure per census tract**	**Annual average sum of person-days of exposure**	**Annual average person-days of exposure per census tract**
	**Mean (SD[d])**			
**Single hazards**				
Extreme heat[a]	133,714 (47,400)	7.4 (3.4)	564,384,773 (201,498,211)	31,171 (19,861)
Wildfire burn zone[b]	7,709 (1,219)	0.4 (2.5)	25,236,989 (4,118,724)	1,394 (7,851)
Wildfire smoke[c]	481,564 (259,819)	26.6 (10.7)	1,976,217,709 (1,087,474,243)	109,147 (60,131)
Extreme heat or wildfire burn zone or wildfire smoke	581,806 (268,376)	32.1 (10.4)	2,397,625,195 (1,122,359,277)	132,422 (65,985)
**Co-occurring hazards**				
Wildfire burn zone and wildfire smoke	2,913 (984)	0.2 (1.1)	8,917,186 (3,074,409)	492 (3,372)
Extreme heat and wildfire burn zone	209 (107)	0.0 (0.1)	699,180 (359,094)	39 (364)
Extreme heat and wildfire smoke	38,214 (30,679)	2.1 (1.1)	159,109,815 (129,945,693)	8,788 (5,771)
Extreme heat, wildfire burn zone, and wildfire smoke	154 (99)	0.0 (0.1)	511,904 (330,385)	28 (290)

[a] Defined based on the local maximum daily temperature equaling or exceeding the warm season 95th percentile and 90°F.

[b] Defined as an active fire/ hot spot identified by MODIS C6.1 MCD41A1 in the census tract.

[c] Defined as wildfire $PM_{2.5}$ concentration over 0 $\mu g/m^3$.

[d] We calculate the standard deviation of tract-days across the census tracts (second and fourth columns). For the average exposure days per census tract, we computed the standard deviation across years (first and third columns).

Table S2.

Sensitivity analysis of annual average prevalence of individual and co-occurring census tract-level climate hazards in 11 Western US states, 2006-2020 when using 5µg/m^3 as the threshold to define wildfire smoke and using two-day (denoted 2D) to define co-exposure events.

	Census tract-day exposures		Person-day exposures	
	**Average total exposure tract-days per year**	**Average exposure days per census tract per year**	**Average total exposure person-days per year**	**Total exposure person-days per census tract per year**
	**Mean (SD)**			
**Individual hazards**				
Wildfire smoke >5µg/m^3	161,283 (167,991)	9 (5)	661,418,741 (700,910,652)	36,530 (24,946)
**Co-occurring hazards**				
Extreme heat and wildfire smoke >5µg/m^3	14,662 (16,560)	1 (0)	60,861,691 (70,261,909)	3,361 (2,440)
Wildfire burn zone and wildfire smoke >5µg/m^3	1,285 (899)	0 (1)	3,819,262 (2,693,796)	211 (1,634)
Extreme heat, wildfire burn zone, and wildfire smoke >5µg/m^3	88 (79)	0 (0)	286,757 (257,456)	16 (148)
**Two-day co-exposure definition**				
Extreme heat (2D)	197,868 (67,190)	11 (5)	835,253,698 (286,001,933)	46,131 (28,696)
Wildfire burn zones (2D)	13,134 (1,799)	1 (4)	43,814,401 (6,318,852)	2,420 (13,050)
Wildfire smoke (2D)	690,931 (304,021)	38 (14)	2,841,718,868 (1,274,428,747)	156,949 (83,056)
Wildfire smoke >5µg/m^3 (2D)	221,407 (200,518)	12 (6)	909,884,776 (836,346,492)	50,253 (32,396)
Extreme heat or wildfire burn zone or wildfire smoke (2D)	822,251 (308,854)	45 (14)	3,393,875,027 (1,293,686,878)	187,445 (91,338)
Extreme heat and wildfire burn zone (2D)	468 (210)	0 (0)	1,598,726 (714,258)	88 (811)
Extreme heat and wildfire smoke >0µg/m^3 (2D)	73,931 (51,943)	4 (2)	308,554,705 (220,062,119)	17,042 (10,919)
Extreme heat and wildfire smoke >5µg/m^3 (2D)	28,495 (29,139)	2 (1)	118,328,725 (123,364,324)	6,535 (4,450)
Wildfire burn zone and wildfire smoke >0µg/ m^3 (2D)	5,649 (1,422)	0 (2)	18,006,301 (4,719,324)	994 (6,251)
Wildfire burn zone and wildfire smoke >5µg/ m^3 (2D)	2,100 (1,281)	0 (1)	6,452,928 (3,972,325)	356 (2,354)
Extreme heat, wildfire burn zone, and wildfire smoke >0µg/ m^3 (2D)	365 (198)	0 (0)	1,247,793 (686,347)	69 (695)
Extreme heat, wildfire burn zone, and wildfire smoke >5µg/ m^3 (2D)	201 (156)	0 (0)	668,768 (529,089)	37 (321)

Table S3.

Annual average statewide person-days of exposure to climate hazards in 11 Western US states, 2006-2020.

If a hazard occurred in a tract, the entire tract was treated as exposed, i.e., we assumed that all individuals within the tract were exposed. Census tract population counts were based on the 2016-2020 American Community Survey (ACS) (via CDC/ATSDR Social Vulnerability Index data).

	Annual average person-days of exposure (millions)						
**State**	Extreme Heat[a]	Wildfire Burn Zone[b]	Wildfire Smoke[c]	Extreme Heat-Wildfire Burn Zone	Extreme Heat-Wildfire Smoke	Wildfire Burn Zone-Wildfire Smoke	Extreme Heat-Wildfire Burn Zone-Wildfire Smoke
AZ	64.1	2.1	101.6	0.11	14.2	0.75	0.08
CA	297.6	8.7	993.6	0.19	72.5	2.7	0.14
CO	48.2	1.0	172.7	0.02	17.8	0.28	0.01
ID	11.1	2.9	77.9	0.05	5.5	1.27	0.04
MT	5.5	1.6	51.8	0.04	3.2	0.73	0.03
NM	21.7	1.1	46.2	0.05	5.6	0.30	0.03
NV	25.6	0.38	60.5	0.03	6.7	0.16	0.03
OR	35.4	3.5	149.4	0.10	12.9	1.3	0.08
UT	23.7	0.94	80.5	0.03	8.6	0.34	0.02
WA	28.4	2.4	218.2	0.04	10.7	0.77	0.03
WY	3.1	0.62	23.8	0.02	1.6	0.28	0.02

[a] Extreme heat (defined based on the local maximum daily temperature equaling or exceeding the warm season 95th percentile and 90°F).

[b] Wildfire burn zones (defined as an active fire/ hot spot identified by MODIS C6.1 MCD41A1 in the census tract).

[c] Wildfire smoke (defined as wildfire $PM_{2.5}$ concentration over 0 $\mu g/m^3$).

Table S4.
Temporal trends of climate hazard person-days by state in 11 Western US states, 2006–2020. Values in the table represent the *p* value for the Mann-Kendall test for trend in person-days of exposure from 2006–2020.

	Exposure						
**State**	Extreme Heat[a]	Wildfire Burn Zone[b]	Wildfire Smoke[c]	Extreme Heat-Wildfire Burn Zone	Extreme Heat-Wildfire Smoke	Wildfire Burn Zone-Wildfire Smoke	Extreme Heat-Wildfire Burn Zone-Wildfire Smoke
AZ	0.198	1.000	0.006	0.060	0.428	0.075	0.029
CA	0.023	0.322	0.060	0.276	0.018	0.322	0.428
CO	0.048	0.921	0.018	0.843	0.322	0.038	0.691
ID	0.621	0.198	0.060	1.000	0.843	0.843	0.519
MT	0.692	0.113	0.428	0.553	0.921	0.767	0.621
NM	0.013	0.018	0.060	0.092	0.018	0.488	0.138
NV	0.048	0.692	0.113	1.000	0.092	0.843	0.920
OR	0.048	0.488	0.038	0.373	0.038	0.198	0.553
UT	0.113	0.322	0.138	0.400	0.322	0.921	0.765
WA	0.322	0.843	0.029	0.921	0.060	0.621	0.692
WY	0.921	0.075	0.138	0.881	0.843	0.166	0.238

[a] Defined ab on the local maximum daily temperature equaling or exceeding the warm season 95th percentile and 90°F.
[b] Defined as an active fire/ hot spot identified by MODIS C6.1 MCD41A1 in the census tract.
[c] Defined as wildfire $PM_{2.5}$ concentration over 0 $\mu g/m^3$.
